# Supplementary material for: Functional rescue and AI analysis of a human inactivating GPCR mutation using a small molecule
Source: EMBO Mol Med. 2026 Jan 8;18(2):725–58. doi: 10.1038/s44321-025-00369-2 (PMC12905377; doi:10.1038/s44321-025-00369-2)
Supplement: Supplementary file 1 — Appendix [file 44321_2025_369_MOESM1_ESM.pdf]

Appendix Figures and Tables for the Manuscript

Functional rescue and AI analysis of a human inactivating GPCR mutation using a small molecule

Debajyoti Das, Amanda Wyatt, Sarath Sivaprasad, Vanessa Wahl, Sen Qiao, Fabien Ectors, Zulfiah M Moosa, Claire L Newton, Mario Fritz, Robert P Millar, Ulrich Boehm

Table of Contents

Appendix Figure S1…...…...…………………………………………………………………..3

Appendix Figure S2…...……………………………………………………………………….4

Appendix Figure S3…...……………………………………………………………………….5

Appendix Figure S4…...……………………………………………………………………….7

Appendix Figure S5…...……………………………………………………………………….8

Appendix Figure S6…...……………………………………………………………………..10

Appendix Figure S7…...……………………………………………………………………..12

Appendix Figure S8…...……………………………………………………………………..13

Appendix Figure S9…...……………………………………………………………………..15

Appendix Figure S10.....……………………………………………………………………..16

Appendix Figure S11.....……………………………………………………………………..18

Appendix Figure S12.....……………………………………………………………………..19

Appendix Figure S13.....……………………………………………………………………..20

Appendix Table S1.......……………………………………………………………………..22

Appendix Table S2.......……………………………………………………………………..23

Appendix Table S3.......……………………………………………………………………..24

Appendix Table S4.......……………………………………………………………………..25

Appendix Table S5.......……………………………………………………………………..26

Appendix Table S6.........……………………………………………………………………………..27

Appendix Table S7.........……………………………………………………………………………..28

Appendix Table S8.........……………………………………………………………………………..29

APPENDIX FIGURES AND FIGURE LEGENDS

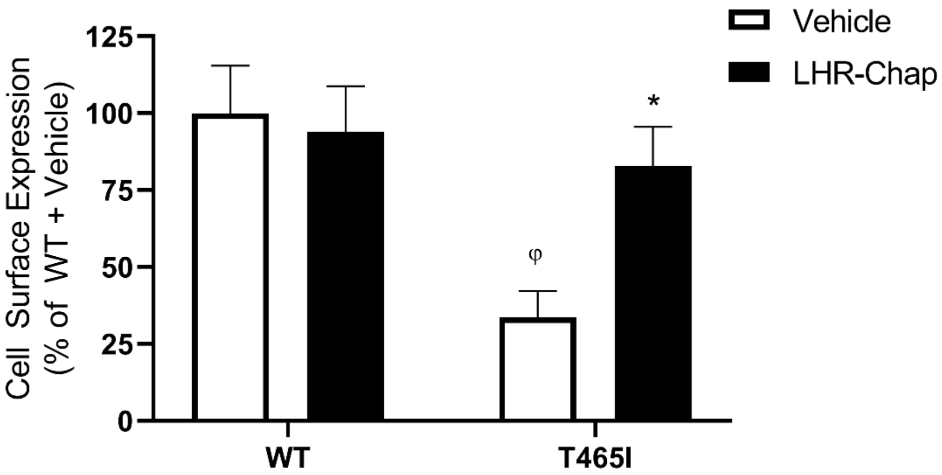

Appendix Figure S1 - Cell surface expression of T465I mLHR is increased upon incubation with LHR-Chap.

Receptor cell surface expression was measured by ELISA targeting the FLAG epitope tag in intact HEK 293T cells transiently expressing FLAG-tagged WT or T465I mLHRs. Cells were treated in the absence (white bars) or presence (black bars) of LHR-Chap (1 µM) for 24 h prior to measurement of receptor expression. Data are presented as a percentage of vehicle-treated WT mLHR expression after subtraction of non-specific signal, measured in empty-vector transfected cells.

Data information: Data are presented as mean ± SEM from 3 independent experiments. $^*P \leq 0.05$, $^{\varphi}P < 0.05$ (one-way ANOVA with Tukey's post-hoc test).

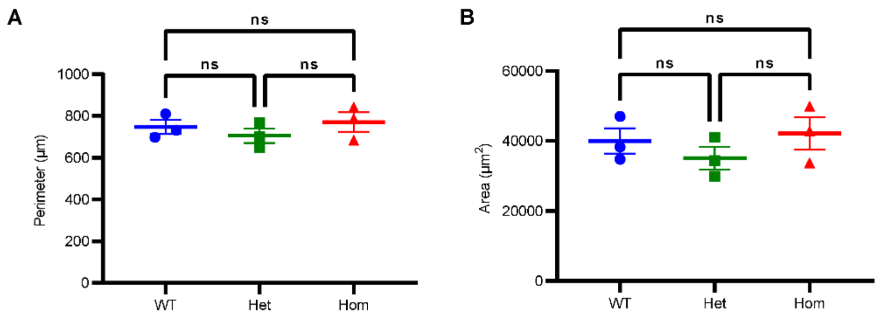

Appendix Figure S2 - The LhrT465I mutation does not affect the dimensions of seminiferous tubules.

A Quantification of the perimeter (boundary) of seminiferous tubules; no difference was observed among WT, heterozygous (Het), and homozygous (Hom) mutant mice. ns indicates lack of statistical significance (*P* = 0.742 for WT vs. Het, *P* = 0.8977 for WT vs. Hom, and *P* = 0.4946 for Het vs. Hom).

B Quantification of the enclosed area of seminiferous tubules; no difference was observed among the groups (*P* = 0.6658 for WT vs. Het, *P* = 0.9198 for WT vs. Hom, and *P* = 0.4541 for Het vs. Hom).

Data information: In (A) and (B), data are presented as mean ± SEM. *$P \leq 0.05$ (one-way ANOVA with Tukey's post-hoc test).

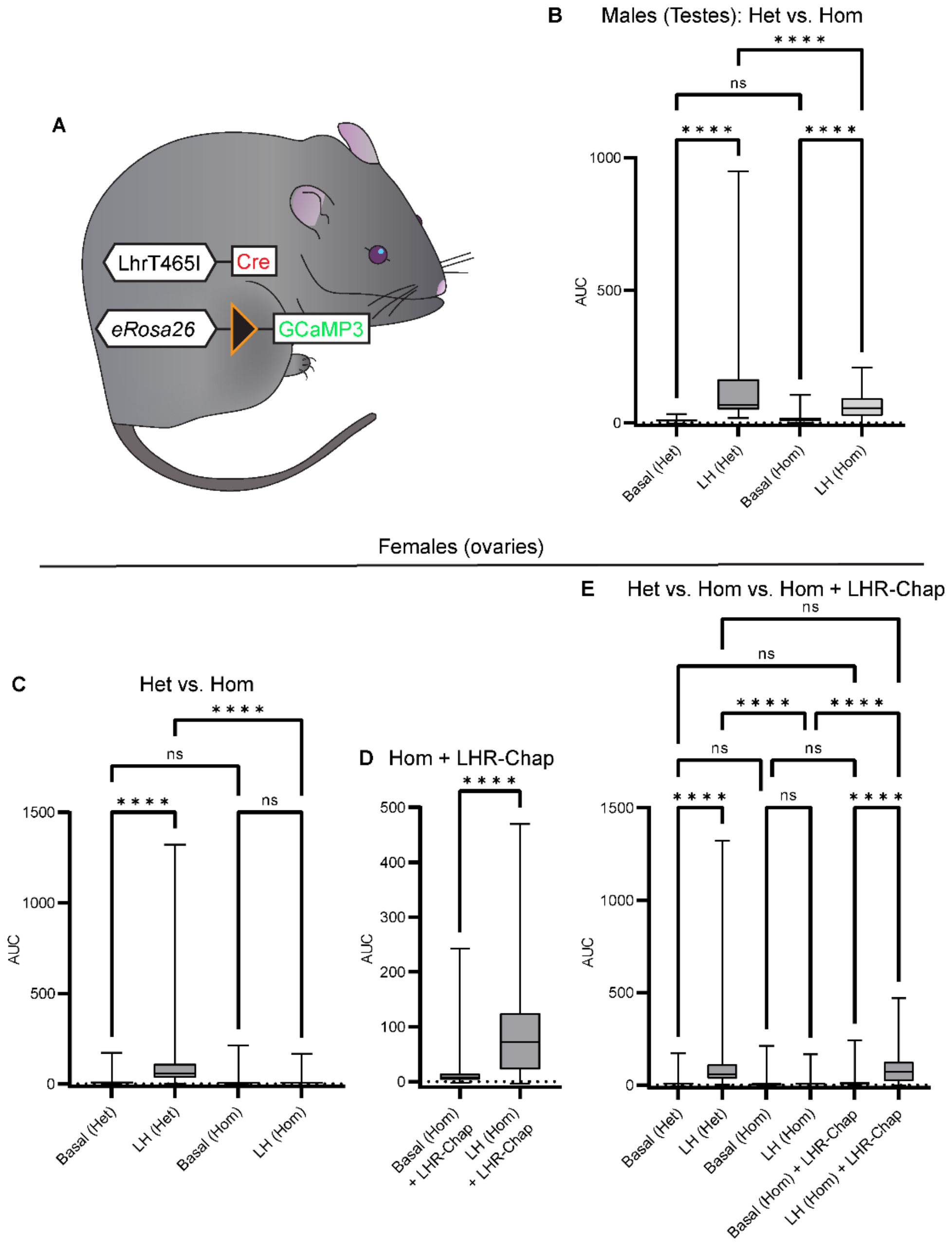

Appendix Figure S3 - Leydig cells of LhrT465I-IC$^{+/+}$/eR26-GCaMP3$^{+/-}$ mice show diminished calcium responses to LH, while the ovarian cells show no response at all.

A LhrT465I-IC$^{+/-}$/eR26-GCaMP3 (Het) and LhrT465I-IC$^{+/+}$/eR26- GCaMP3 (Hom) mice were generated by crossing LhrT465I-IC$^{+/-}$ and LhrT465I-IC$^{+/+}$ male mice with eR26-GCaMP3 female mice, respectively. In these mice, all cells expressing *Lhcgr* also express Cre, which excises the stop signal upstream of GCaMP3. Hence, all cells expressing the luteinizing hormone receptor (LHR) also express GCaMP3.

B Box-and-whisker plot showing quantification of the area under curve (AUC) for the calcium responses of all Leydig cells before (“Basal”) and during LH application, for Het and Hom male mice. $n = 278$ cells from 3 Het mice, 175 cells from 3 Hom mice. First and third quartiles are shown by the box edges; median is shown by the solid line inside the box. Asterisks depict statistical significance (****$P < 0.0001$ for Basal [Het] vs. LH [Het], LH [Het] vs. LH [Hom], and Basal [Hom] vs. LH [Hom]); ns ($P > 0.9999$ for Basal [Het] vs. Basal [Hom]) indicates lack of statistical significance.

C Box-and-whisker plot showing quantification of the AUC for the calcium responses of all ovarian cells before and during LH application, for Het and Hom female mice. $n = 401$ cells from 5 Het mice, 203 cells from 5 Hom mice. ****$P < 0.0001$ for Basal [Het] vs. LH [Het] and LH [Het] vs. LH [Hom]; ns, $P = 0.7732$ for Basal [Het] vs. Basal [Hom] and $P > 0.9999$ for Basal [Hom] vs. LH [Hom].

D Box-and-whisker plot showing quantification of the AUC for the calcium responses of all ovarian cells from LHR-Chap-treated Hom mice (****$P < 0.0001$).

E Combined box-and-whisker plot showing quantification of the AUC of all ovarian cells from Het, Hom, and LHR-Chap-treated Hom female mice. $n = 401$ cells from 5 Het mice, 203 cells from 5 Hom mice, and 391 cells from 3 LHR-Chap-treated Hom mice. ****$P < 0.0001$ for LH [Hom] vs. LH [Hom] + LHR-Chap; ns, $P > 0.9999$ for Basal [Het] vs. Basal [Hom] + LHR-Chap, for Basal [Hom] vs. Basal [Hom] + LHR-Chap, and for LH [Het] vs. LH [Hom] + LHR-Chap.

Data information: In (B), (C), and (E), data are presented as box-and-whisker plots. ****$P < 0.0001$ (two-way ANOVA with Bonferroni post hoc test).

In (D), data are presented as box-and-whisker plots. ****$P < 0.0001$ (paired two-tailed Student’s t-test).

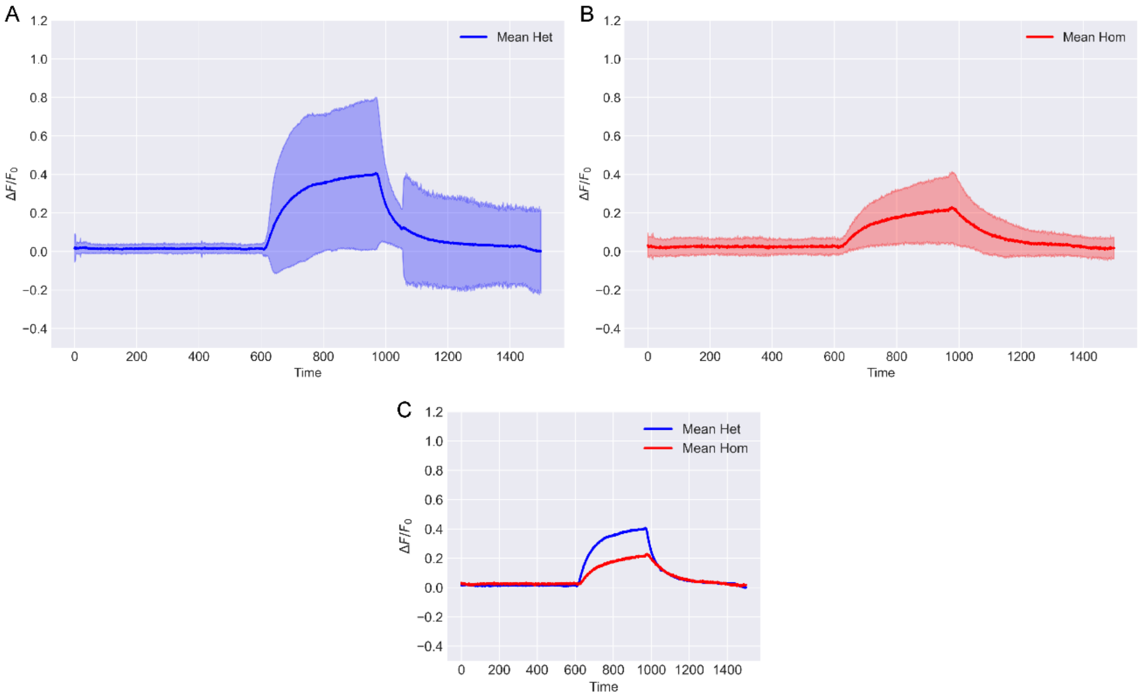

Appendix Figure S4 - Time-course analysis of change in ΔF/F0 for male groups.

A The plot shows the mean (solid line) and standard deviation (shaded area) for calcium profiles of control Het Leydig cells.

B Mean (solid line) and standard deviation (shaded area) for calcium profiles of mutant Hom Leydig cells.

C Comparison of the mean calcium traces between Het and Hom groups. Mutants exhibit distinct signal deviations compared to controls, highlighting the effect of the mutation.

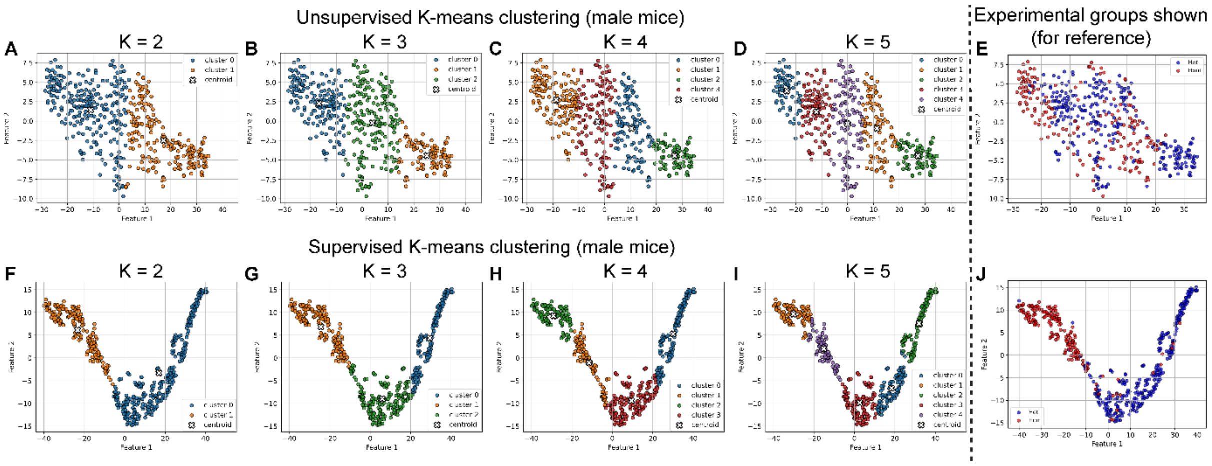

Appendix Figure S5 - K-means clustering of t-SNE feature space reveals distinct clusters for Leydig cells.

A–D Unsupervised K-means clustering, following t-SNE projection, for control Het and Hom Leydig cells. The K-means clustering results, although not immediately evident from the overall statistics, reveal that the algorithm indeed identifies subpopulations that are more homogeneous in their biological composition. For example, at K = 2 (silhouette score: 0.539) (A), the two clusters show moderate differences in composition, with one cluster being approximately 65% Hom, suggesting an initial stratification. When K is increased to 3 (average silhouette score: 0.5359) (B), one cluster becomes notably enriched in Hom cells (77% Hom), while another is almost evenly split (49% Het vs. 51% Hom), indicating emerging separation of signaling types. At K = 4 (average silhouette score: 0.4781) (C), the data further resolve into sub-clusters, with cluster 2 showed a striking 91% Hom composition; this confirms that the t-SNE approach for 2D projection can successfully capture local clustering tendencies resulting from the LHR mutation. At K = 5 (silhouette score: 0.4743) (D), a cluster dominated by control Het cells emerges (approximately 85% Het), while other Hom clusters maintain strong homogeneity (over 91% Hom in another cluster).

E t-SNE projection showing composition of the unsupervised clusters, i.e. proportion of cells in the Het or Hom group.

F–I Supervised (trained AI model) K-means clustering, following t-SNE projection, for control Het and Hom Leydig cells. At K = 2 (E) itself, the two clusters showed strong enrichment, indicating accurate detection of Hom-specific calcium profile characteristics by the AI model. With K = 3 (F), a clear

structure begins to emerge: one cluster was highly enriched for Het cells (94.7%), and another predominantly composed of Hom cells (91.3%). This trend became more pronounced at K = 4 (G), where two clusters were highly specific to either Het or Hom classes (91% purity), while a third showed near-equal representation, potentially capturing ambiguous or intermediate signaling profiles. The best silhouette score (0.557) was observed at K = 4, indicating well-separated, cohesive clusters. Increasing to K = 5 (H) led to the emergence of all high-purity clusters (91%), three of Hom-dominant and two distinct control Het-dominant clusters (93%), suggesting further refinement of response subtypes.

J t-SNE projection showing composition of the supervised clusters, i.e. proportion of cells in the Het or Hom group.

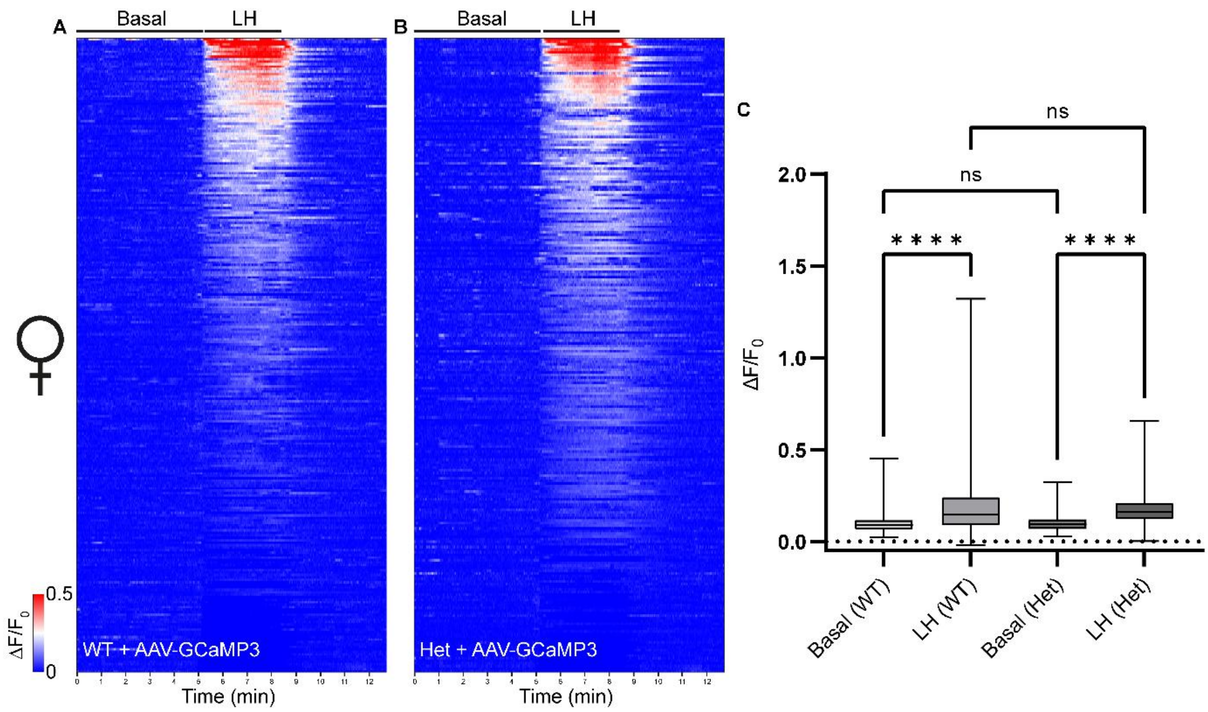

Appendix Figure S6 - Ovarian cells of WT and Het female mice show similar calcium responses to LH.

A Heatmap showing collected normalized fluorescence intensities from 256 ovarian cells of AAV-GCaMP3-microinjected LhrT465I-IC$^{-/-}$ (WT) mice (pooled from 4 mice). The heatmap consists of 3 sections. The first part indicates the time from the start of the experiment up to LH application. This part of the heatmap shows the spontaneous/basal activity of the ovarian cells. The second part (marked by "LH") indicates the bath application of 1 IU/ml LH. The third part indicates washing of the LH with recording buffer. Cells in the heatmap have been represented in the descending order of fluorescence intensities of their Ca^{2+} responses to LH. Scale of the changes in fluorescence intensities in the heatmap are indicated by the color-coded bar.

B Heatmap showing collected normalized fluorescence intensities from 242 ovarian cells of AAV-GCaMP3-microinjected LhrT465I-IC$^{+/-}$ (Het) mice (pooled from 4 mice).

C Box-and-whisker plot showing quantification of the total change in fluorescence intensities for all cells before ("Basal") and during LH application, for WT and Het mice. Asterisks depict statistical significance (****$P < 0.0001$ for Basal [WT] vs. LH [WT] and Basal [Het] vs. LH [Het]), ns ($P > 0.9999$

for Basal [WT] vs. Basal [Het] and $P = 0.4782$ for LH [WT] vs. LH [Het]) indicates lack of statistical significance. For WT, $n = 256$ cells (from 4 mice). For Het, $n = 242$ cells (from 4 mice).

Data information: In (C), data are presented as box-and-whisker plots. ****$P < 0.0001$ (two-way ANOVA with Bonferroni post hoc test).

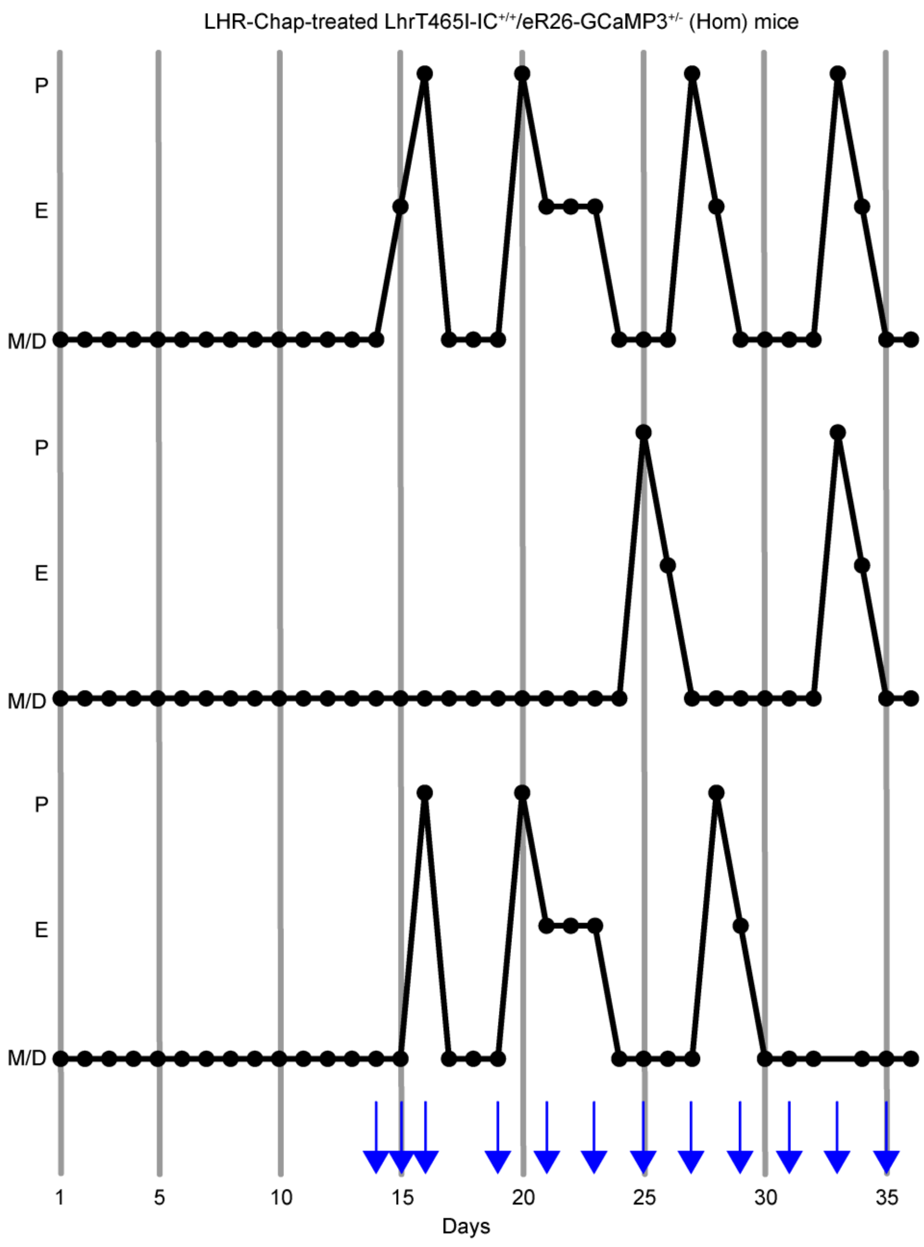

Appendix Figure S7 - LHR-Chap administration regularizes estrous cycling in LhrT465I-IC$^{+/+}$/eR26-GCaMP3$^{+/-}$ (Hom) females.

Estrous cycle stages were recorded for 14 days for adult Hom females (untreated). They were then i.p.-injected with 25 mg/kg LHR-Chap on the days indicated by the blue arrows (starting on the 14th day). As observed for LhrT465I-IC$^{+/+}$ female mice, LHR-Chap administration caused a relative regularization of the estrous cycles, with proestrus stages being detected. P, proestrus; E, estrus; M/D, metestrus/diestrus

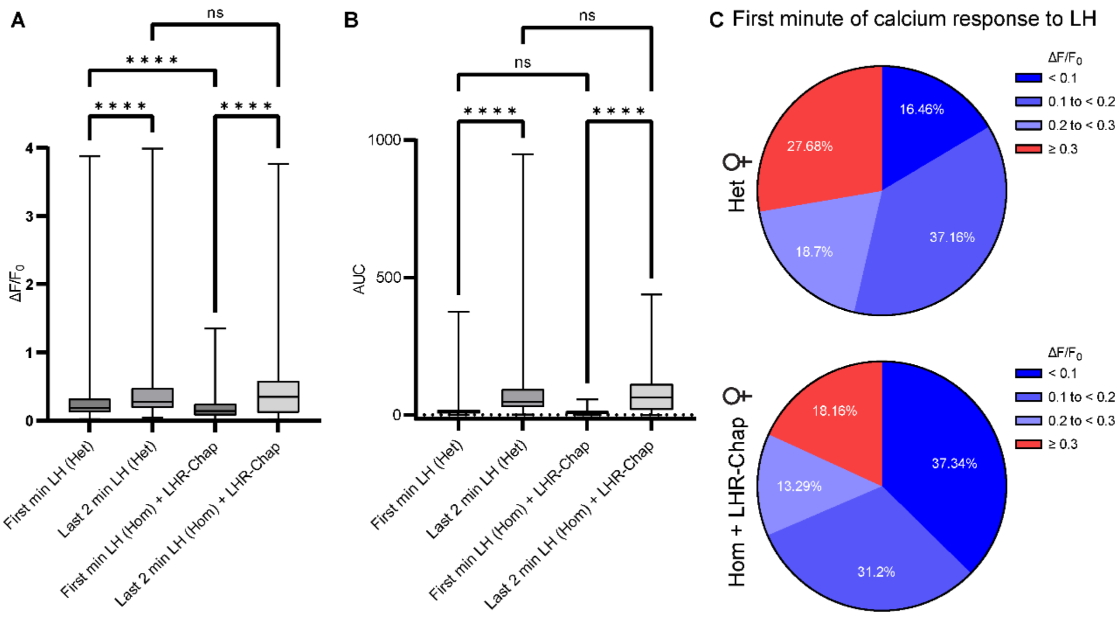

Appendix Figure S8 - Calcium response of ovarian cells from LhrT465I-IC$^{+/+}$/eR26-GCaMP3$^{+/-}$ (Hom) females to LH application is delayed.

A Box-and-whisker plot showing quantification of the total change in fluorescence intensities for all ovarian cells during the first and the last 2 min of LH application (total application duration: 3 min), for Het ($n = 401$, $N = 5$) and LHR-Chap-treated Hom ($n = 391$, $N = 3$) mice. Analyzing the calcium responses separately for these two time-windows reveals the delay in the response of the ovarian cells from LHR-Chap-treated Hom mice compared to the Het response. First and third quartiles are shown by the box edges; median is shown by the solid line inside the box. Asterisks depict statistical significance (****P < 0.0001 for First min LH [Het] vs. Last 2 min LH [Het] and for First min LH [Hom] + LHR-Chap vs. Last 2 min LH [Hom] + LHR-Chap, and First min LH [Het] vs. First min LH [Hom] + LHR-Chap); ns (P > 0.9999 for Last 2 min LH [Het] vs. Last 2 min LH [Hom] + LHR-Chap) indicates lack of statistical significance.

B Box-and-whisker plot showing quantification of the total change in area under curve (AUC) for all ovarian cells during the first and the last 2 min of LH application, for Het and LHR-Chap-treated Hom mice. ****P < 0.0001 for First min LH [Het] vs. Last 2 min LH [Het] and First min LH [Hom] + LHR-Chap vs. Last 2 min LH [Hom] + LHR-Chap. ns, P > 0.9999 for First min LH [Het] vs. First min LH [Hom] + LHR-Chap and for Last 2 min LH [Het] vs. Last 2 min LH [Hom] + LHR-Chap.

C Pie charts showing the percentage of active ($\geq 0.1\ \Delta F/F_0$) and inactive cells ($< 0.1\ \Delta F/F_0$) during the first min of LH application, for Het and LHR-Chap-treated Hom mice. This further resolves the difference in total change in fluorescence intensities observed between these two groups in (A). Therefore, during the first min of LH application, 37.34% cells are inactive for the LHR-Chap-treated Hom mice, whereas only 16.46% cells are inactive for the Het mice, contributing to the overall delay in response.

Data information: In (A) and (B), data are presented as box-and-whisker plots. ****$P < 0.0001$ (two-way ANOVA with Bonferroni post hoc test).

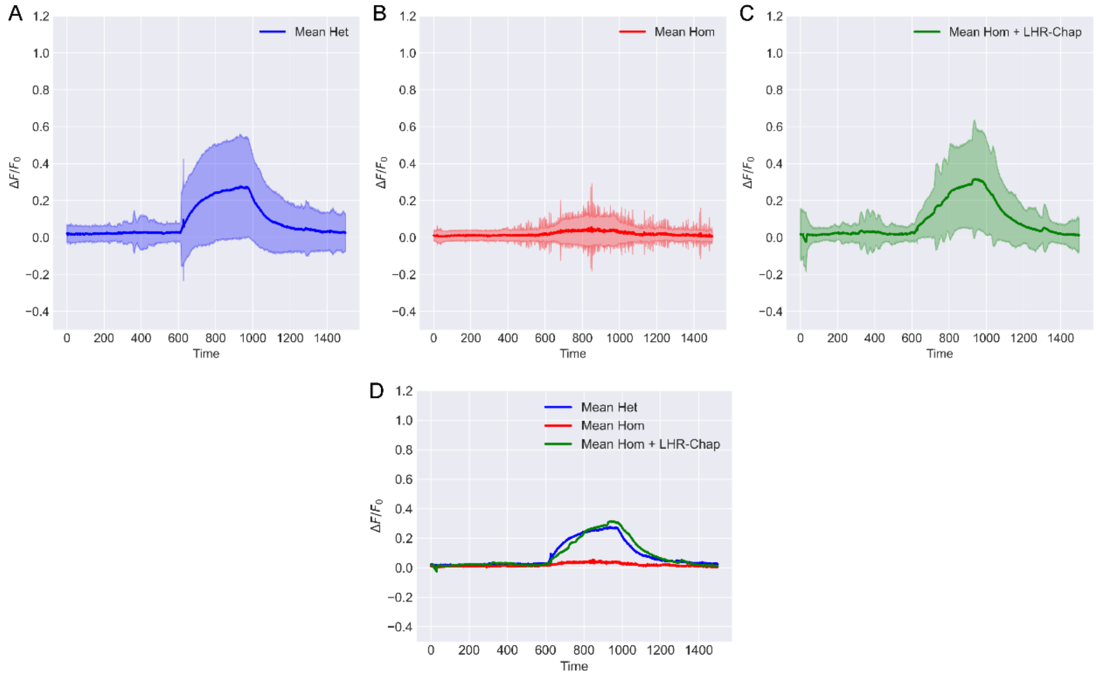

Appendix Figure S9 - Time-course analysis of change in $\Delta F/F_0$ for female groups.

A The plot displays the mean (solid line) and standard deviation (shaded area) for calcium profiles of control Het ovarian cells.

B Mean (solid line) and standard deviation (shaded area) for calcium profiles of mutant Hom ovarian cells.

C Mean (solid line) and standard deviation (shaded area) for calcium profiles of LHR-Chap-treated (rescued) Hom cells. Rescued mutant cells exhibit similar trends compared to control Het samples, indicating a potential effect of treatment.

D Comparison of the mean calcium traces between Het and Hom groups. Mutants exhibit distinct signal deviations compared to controls, highlighting the effect of the mutation.

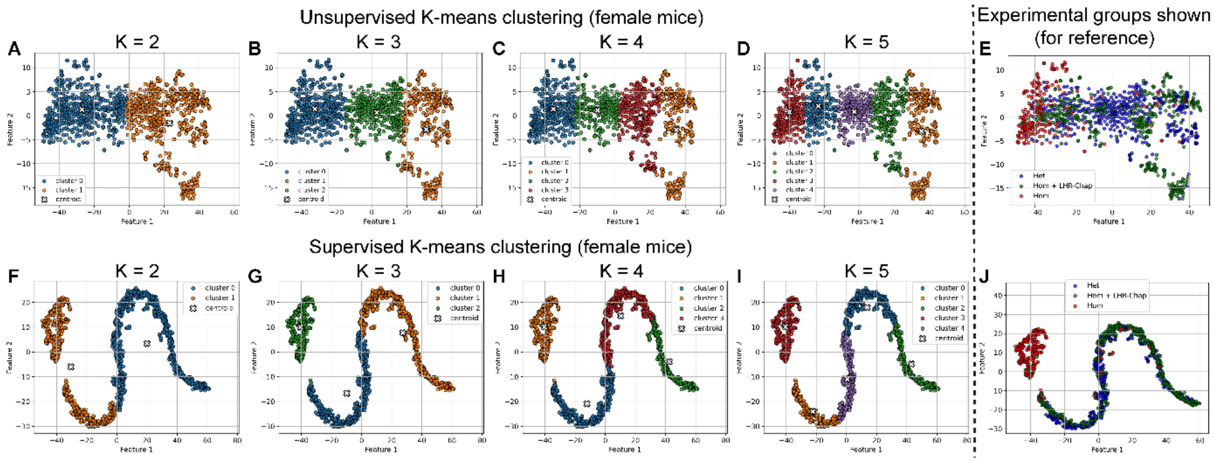

Appendix Figure S10 - K-means clustering of t-SNE feature space reveals distinct clusters for ovarian cells.

A–D Unsupervised K-means clustering for control Het, Hom, and LHR-Chap-treated Hom ovarian cells. At K = 2 (A), the algorithm separated the data into two large clusters, one of which contained a large proportion of untreated Hom cells (approximately 40%), while the other had a more even mixture of Het and treated Hom cells. At K = 3 (B), a more meaningful biological structure emerges: one cluster was predominantly composed of LHR-Chap-treated Hom cells (nearly 60%), another of untreated Hom cells (about 50%), and a third enriched in Het cells (around 65%). This configuration indicated that the algorithm had started to isolate the signaling phenotypes that characterize each condition. At K = 4 (C), the clustering results further reinforced this trend. Distinct clusters emerged that were enriched for each of the three groups: one was composed of 60% untreated Hom cells, while other groups were predominantly composed of the other two classes. With K = 5 (D), one cluster became highly enriched in untreated Hom cells (over 75%), while another showed a strong Het majority (around 70%), and a separate cluster was again dominated by treated Hom cells (approximately 55%). These patterns suggest that the LHR-Chap treatment shifts the calcium signaling pattern of Hom cells closer to that of the control Het group, while still maintaining some of the original distinctive features.

E t-SNE projection showing composition of the unsupervised clusters, i.e. proportion of cells in the Het, untreated Hom, and LHR-Chap-treated Hom groups.

F–I Supervised (trained AI model) K-means clustering for all groups. For K = 2 (silhouette score: 0.594) (E), one cluster contained a mixture of control Het and LHR-Chap-treated Hom cells, with relatively

low representation of untreated Hom cells, while the other was enriched in untreated Hom cells (43% Hom vs. 27% control and 30% LHR-Chap-treated Hom). As K was increased to 3 (silhouette score: 0.564) (F), one cluster becomes predominantly composed of untreated Hom cells, whereas the other two are composed almost exclusively of a combination of control and treated cells. At K = 4 (silhouette score: 0.508) (G) and K = 5 (silhouette score: 0.509) (H), this separation is further refined: distinct clusters emerge that are pure in mutant content (up to 74%) and others dominated by control and treated cells (with upto 90% control Het or LHR-Chap-treated Hom cells in some clusters).

J t-SNE projection showing composition of the supervised clusters, i.e. proportion of cells in the Het, Hom, and LHR-Chap-treated Hom groups. Across all tested values, the unsupervised clusters consistently revealed that untreated mutant cells group apart from both control Het and LHR-Chap-treated cells.

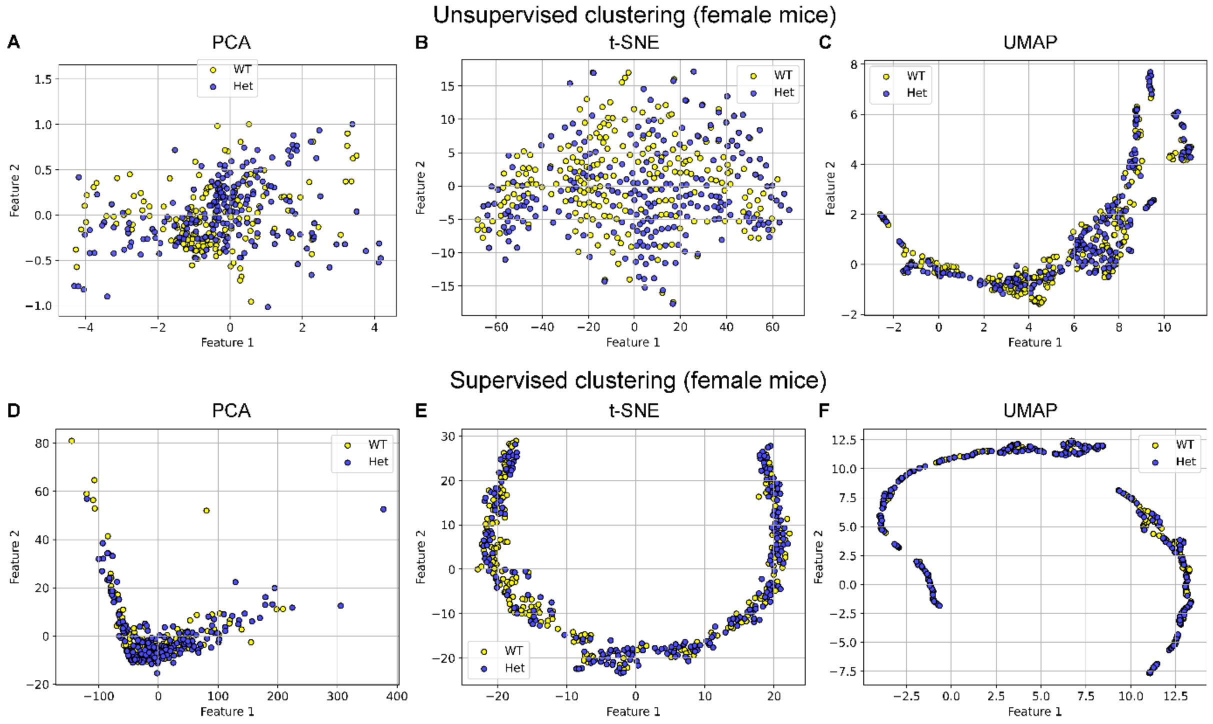

Appendix Figure S11 - AI cannot differentiate between WT and Het ovarian cells.

A, B, C PCA (A), t-SNE (B), and UMAP (C) representations of unsupervised clustering of ovarian cells from wild-type LhrT465I-IC$^{-/-}$ (WT) and LhrT465I-IC$^{+/-}$ (Het) female mice, based on their calcium profiles.

D, E, F PCA (D), t-SNE (E), and UMAP (F) representations of supervised (AI model) clustering of ovarian cells from WT and Het female mice, based on their calcium profiles.

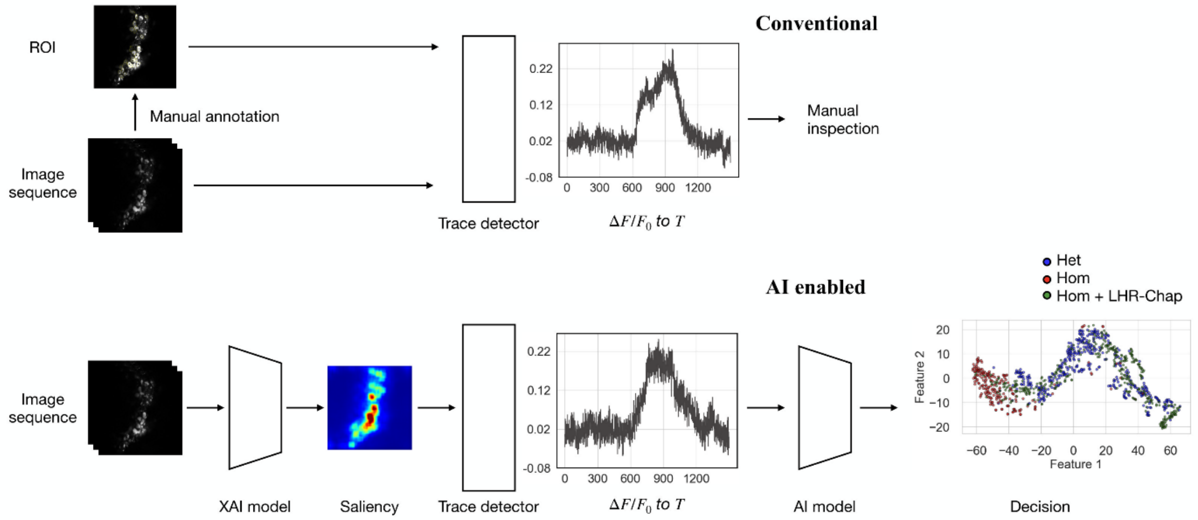

Appendix Figure S12 - Model diagram comparing the AI-enabled workflow proposed here to the conventional image processing pipeline.

The top row illustrates the traditional approach where regions of interest (ROIs) are manually annotated on raw microscopy images, followed by trace extraction and visual inspection of calcium dynamics ($\Delta F/F_0$) to infer spontaneous activity as well as response to LH application.

The bottom row depicts the proposed AI-enabled workflow, for which we provide an initial toolkit here. Here, an explainable AI (XAI) model first classifies the full-field image and generates a saliency map that identifies relevant ROIs automatically. Traces can then be extracted from these model-selected regions, enabling both manual inspection and automated downstream classification. This step will be validated in future work. A second AI model processes these traces and maps the sample into a low-dimensional feature space, revealing structured groupings among control (Het), mutant (Hom), and pharmacologically rescued (LHR-Chap-treated Hom) cells.

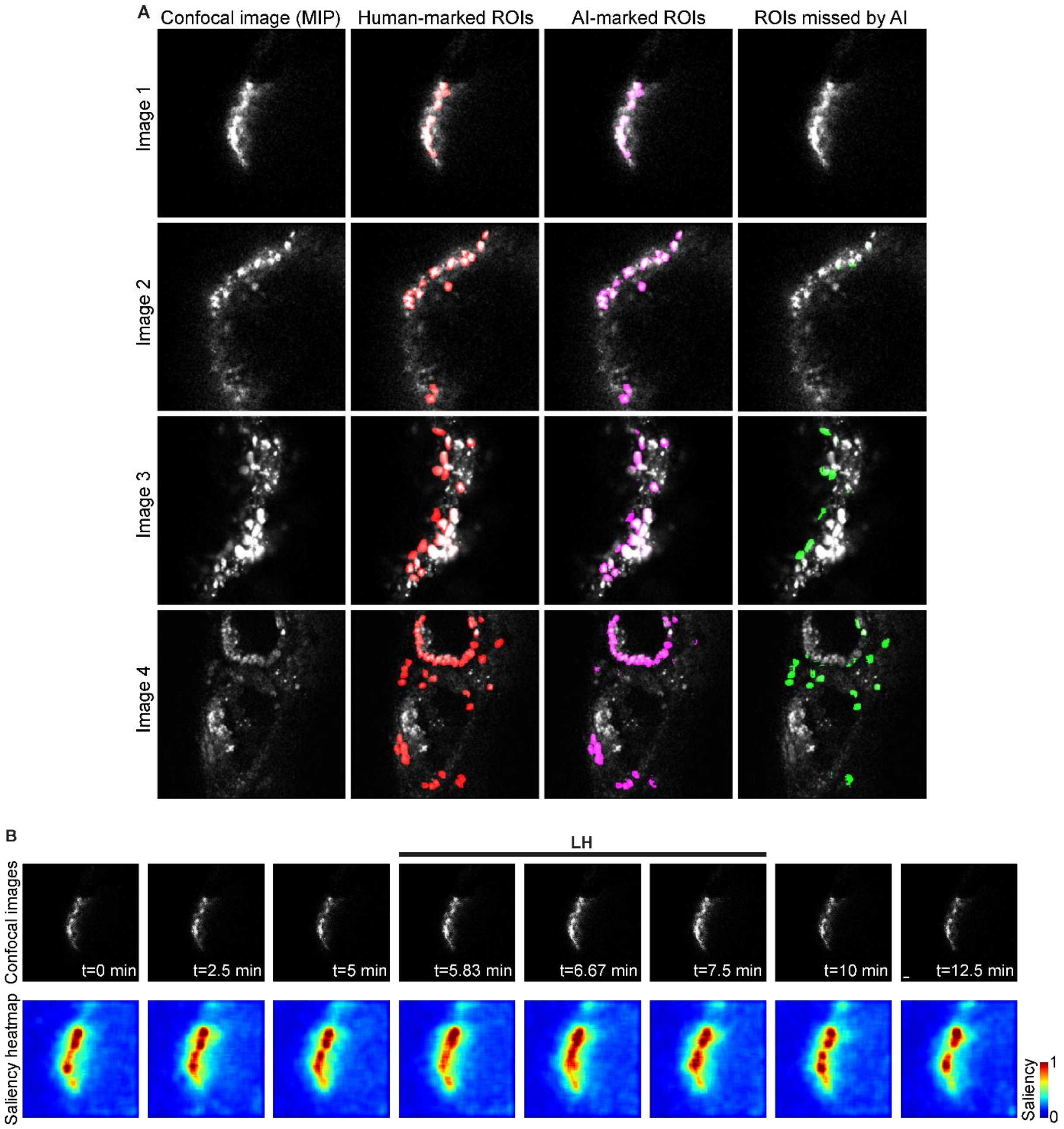

Appendix Figure S13 - Saliency of raw confocal images detected and used by the AI model. A Four representative confocal images of ovarian slices and their processing to identify regions of interest have been shown. The first column shows raw representative maximum intensity projections (MIPs) of time-series confocal recordings. The second column shows ROIs manually marked by a human experimenter on these MIP images. The third column shows a composite overlay (for the entire time-series) of ROIs (salient features) identified by the AI model. The fourth column shows ROIs marked by the experimenter that were missed by the AI model. These examples highlight the range of efficiency of the AI model. These overlays illustrate substantial overlap between human- and AI-selected ROIs, with localized disagreement highlighting areas for refinement and model improvement. B For representative Image 1,

snapshots at specific time-points across the confocal recording and their corresponding individual saliency heatmaps have been shown. LH bath application was started at t=5 min and washing was started at t=7.5 min; hence, t=5.83 min, t=6.67 min, and t=7.5 min show the calcium response to LH. Saliency (probabilistic) was plotted as a heatmap, with 0 being the lowest and 1 the highest. All saliency heatmaps are normalized to themselves to avoid compromised detection at certain time-points due to low absolute fluorescence intensity. Scale bar (confocal images): 20 μm

APPENDIX TABLES AND TABLE LEGENDS

Appendix Table S1 - Unsupervised t-SNE clustering results for Leydig cells for different K values.

K	Silhouette score	Cluster	Cluster size	Het (%)	Hom (%)
2	0.539	0	259	59.1	40.9
		1	195	64.6	35.4
3	0.5359	0	165	50.9	49.1
		1	100	77	23
		2	189	62.4	37.6
4	0.4781	0	108	42.6	57.4
		1	132	47	53
		2	81	91.4	8.6
		3	133	72.9	27.1
5	0.4743	0	66	15.2	84.8
		1	96	41.7	58.3
		2	80	91.2	8.8
		3	100	75	25
		4	112	72.3	27.7

This table shows K Nearest Neighbor clustering of calcium profiles of Leydig cells after dimensionality reduction using t-SNE. The columns describe cluster identity, size (number of cells), silhouette score, and percentages of control Het and mutant Hom cells in each cluster. The silhouette scores (0.45–0.54) with moderate cluster sizes (compared to full-dimensional clustering [Appendix Table S2]) indicate improved separation, with certain clusters being enriched in Hom or Het cells. This suggests that t-SNE captures nonlinear structure that better discriminates genotype-driven signaling states. Taken together, the unsupervised embedding highlights intrinsic heterogeneity with partial segregation of mutant Hom and control Het groups.

Appendix Table S2 - Unsupervised full-dimensional clustering results for Leydig cells for different K values.

K	Silhouette score	Cluster	Cluster size	Het (%)	Hom (%)
2	0.6802	0	384	55.2	44.8
		1	70	95.7	4.3
3	0.6904	0	382	55.2	44.8
		1	71	94.4	5.6
		2	1	100	0.0
4	0.6765	0	78	87.2	12.8
		1	373	55.8	44.2
		2	2	100	0.0
		3	1	100	0.0
5	0.5313	0	346	57.8	42.2
		1	2	100	0.0
		2	48	100	0.0
		3	1	100	0.0
		4	57	49.1	50.9

This table shows results from K Nearest Neighbor-based clustering of Leydig cell calcium profiles using all dimensions without supervision. The columns list cluster number, cluster size (number of cells), silhouette score, and percentages of control Het and mutant Hom cells in each cluster. The results indicate moderate separation despite silhouette values ranging 0.53–0.69, due to many pure clusters containing only 1 or 2 cells. Only a few moderately sized clusters (around 40–80 cells) were enriched in Het cells. This approach offers very biologically insight, with most of the cells from both groups forming a single cluster even at K = 5, indicating that unsupervised full dimensional analysis is not suitable in this context.

Appendix Table S3 - Supervised (trained AI model) t-SNE clustering results for Leydig cells for different K values.

K	Silhouette score	Cluster	Cluster size	Het (%)	Hom (%)
2	0.4705	0	200	94	6
		1	254	7.1	92.9
3	0.584	0	95	95.2	4.8
		1	204	3.2	96.8
		2	155	90.6	9.4
4	0.6114	0	95	95.7	4.3
		1	87	31.8	68.2
		2	105	1	99
		3	167	94.6	5.4
5	0.6658	0	95	96.6	3.4
		1	64	1.1	98.9
		2	105	96.7	3.3
		3	87	89.6	10.4
		4	103	13.2	86.8

This table shows supervised (AI model) K Nearest Neighbor classification using t-SNE embedding (reduced dimensions) of Leydig cell calcium profiles. The columns list silhouette values (0.47–0.67), cluster number, cluster size (number of cells), and percentages of control Het and mutant Hom cells in each cluster, demonstrating near-perfect separation, with clusters containing > 95% Het or Hom cells. These results reflect a strong alignment between the embedding and genotype-specific calcium dynamics. Therefore, AI-based supervised nonlinear feature extraction isolates robust differences in calcium signaling signatures between Het and Hom groups. Overall, this method offers accurate classification based on effect of the LHR mutation on calcium signaling.

Appendix Table S4 - Supervised (trained AI model) full-dimensional clustering results for Leydig cells for different K values.

K	Silhouette score	Cluster	Cluster size	Het (%)	Hom (%)
2	0.6749	0	40	100	0.0
		1	414	57.7	42.3
3	0.5735	0	275	92	8
		1	157	2.5	97.5
		2	22	100	0.0
4	0.5837	0	1	100	0.0
		1	274	92	8
		2	23	100	0.0
		3	156	1.9	98.1
5	0.6059	0	251	90.8	9.2
		1	154	1.9	98.1
		2	5	100	0.0
		3	1	100	0.0
		4	43	97.7	2.3

This table summarizes results from supervised (AI model) K Nearest Neighbor clustering performed on full dimensional data for Leydig cell calcium profiles. The high silhouette scores (0.57–0.67) and near-homogeneous cluster compositions reveal highly enriched Het (> 90%) or Hom (> 90–100%) clusters, with only a few mixed clusters. Therefore, full dimensional analysis of supervised feature-learning accurately captures discriminative features of calcium dynamics between control Het and mutant Hom groups, allowing their robust classification.

Appendix Table S5 - Unsupervised t-SNE clustering results for ovarian cells for different K values.

K	Silhouette score	Cluster	Cluster size	Het (%)	Hom (%)	LHR-Chap-treated Hom (%)
2	0.6247	0	474	29.7	40.1	30.2
		1	521	49.9	2.5	47.6
3	0.5368	0	369	22.2	49.9	27.9
		1	332	38	3	59
		2	294	65.6	3.1	31.3
4	0.4873	0	304	17.1	58.9	24
		1	245	42	2	55.9
		2	203	58.6	5.4	36
		3	243	52.3	3.3	44.4
5	0.4662	0	184	34.2	21.8	44
		1	234	43.2	2.1	54.7
		2	216	43.5	3.7	52.8
		3	195	10.8	75.4	13.8
		4	166	73.5	1.8	24.7

This table shows results from unsupervised K Nearest Neighbor clustering of t-SNE-reduced features of ovarian cell calcium profiles. The silhouette scores (0.44–0.62) are higher than those obtained with unsupervised full dimensional analysis (Appendix Table S6), reflecting better cluster compactness. Clusters display moderate enrichment of either untreated mutant Hom (> 75%) or control Het and LHR-Chap-treated Hom cells, with intermediate clusters hinting at partial functional rescue at the single-cell level. Therefore, dimensionality reduction captures LHR-Chap treatment-dependent divergence (from untreated Hom cells) more effectively than full raw features.

Appendix Table S6 - Unsupervised full dimensional clustering results for ovarian cells for different K values.

K	Silhouette score	Cluster	Cluster size	Het (%)	Hom (%)	LHR-Chap-treated Hom (%)
2	0.5667	0	760	39.7	26.2	34.1
		1	235	42.1	1.7	56.2
3	0.5633	0	753	39.6	26.4	34
		1	1	100	0.0	0.0
		2	241	42.3	1.7	56
4	0.3933	0	315	47	3.8	49.2
		1	1	100	0.0	0.0
		2	516	34.9	36.6	28.5
		3	163	44.2	1.2	54.6
5	0.3749	0	460	29.1	41.1	29.8
		1	55	45.5	0.0	54.5
		2	172	41.3	2.3	56.4
		3	1	100	0.0	0.0
		4	307	55.4	3.3	41.4

This table reports unsupervised full dimensional K Nearest Neighbor clustering of ovarian cell calcium profiles. Clusters are annotated with silhouette scores (0.37–0.57), sizes (number of cells), and percentages of control Het, untreated mutant Hom, and LHR-Chap-treated Hom cells. Enrichment of clusters beyond 60% was not observed for any group (excluding the single-cell cluster). The results show that unsupervised full dimensional analysis yields partially segregation, with LHR-Chap-treated Hom cells often clustering with control Het cells rather than untreated Hom cells. Despite limited resolution, this indicates that LHR-Chap-treatment predominantly rescues calcium signaling profiles of Hom cells.

Appendix Table S7 - Supervised (trained AI model) t-SNE clustering results for ovarian cells for different K values.

K	Silhouette score	Cluster	Cluster size	Het (%)	Hom (%)	LHR-Chap-treated Hom (%)
2	0.4809	0	417	25.7	45.6	28.8
		1	578	50.9	2.2	46.9
3	0.5082	0	402	39.8	2.5	57.7
		1	243	1.6	77.8	20.6
		2	350	67.7	1.1	31.1
4	0.5622	0	289	61.6	1.4	37
		1	282	47.9	3.9	48.2
		2	225	1.3	83.1	15.6
		3	199	42.7	0.5	56.8
5	0.5633	0	246	38.6	4.1	57.3
		1	166	48.8	2.4	48.8
		2	169	43.2	0.6	56.2
		3	220	0.9	84.5	14.5
		4	194	77.3	1	21.6

This table gives details of supervised (AI model) K Nearest Neighbor clustering performed on t-SNE embedding of ovarian cell calcium profiles. High silhouette scores (0.48–0.56) indicate compact and meaningful separation of clusters, most of which are strongly enriched (> 70%) in either untreated Hom cells or control Het and LHR-Chap-treated Hom cells. Strikingly, most LHR-Chap-treated Hom cells co-cluster with Het rather than untreated Hom cells, demonstrating effective functional rescue at the single-cell level, with very few exceptions. Overall, supervised t-SNE clustering provides the clearest resolution of treatment effect in Hom cells.

Appendix Table S8 - Supervised (trained AI model) full-dimensional clustering results for ovarian cells for different K values.

K	Silhouette score	Cluster	Cluster size	Het (%)	Hom (%)	LHR-Chap-treated Hom (%)
2	0.6662	0	773	39.7	26	34.3
		1	222	42.3	0.9	56.8
3	0.5891	0	296	39.5	2.7	57.8
		1	89	36	0.0	64
		2	610	41.3	32	26.7
4	0.5763	0	314	46.5	3.2	50.3
		1	156	42.9	0.6	56.4
		2	522	35.8	36.8	27.4
		3	3	33.3	0.0	66.7
5	0.5777	0	321	57.9	3.1	38.9
		1	80	33.8	0.0	66.2
		2	428	26.6	44.6	28.7
		3	3	33.3	0.0	66.7
		4	163	44.8	1.2	54

This table presents details of supervised (AI model) full-dimensional K Nearest Neighbor clustering of ovarian cell calcium profiles. The clusters show high silhouette scores of 0.57–0.67, with some showing clear enrichment of control Het- as well as LHR-Chap-treated Hom cells. Therefore, full dimensional supervised analysis clustering robustly demonstrates LHR-Chap-mediated restoration of calcium signaling and LH response to a control Het-like state. However, in contrast to t-SNE embedded supervised clustering of ovarian cells (Appendix Table S7), clusters enriched in untreated Hom cells (> 70%) were lacking.
