# Supplementary figures and images for: Functional rescue and AI analysis of a human inactivating GPCR mutation using a small molecule
Source: EMBO Mol Med. 2026 Jan 8;18(2):725–58. doi: 10.1038/s44321-025-00369-2 (PMC12905377; doi:10.1038/s44321-025-00369-2)

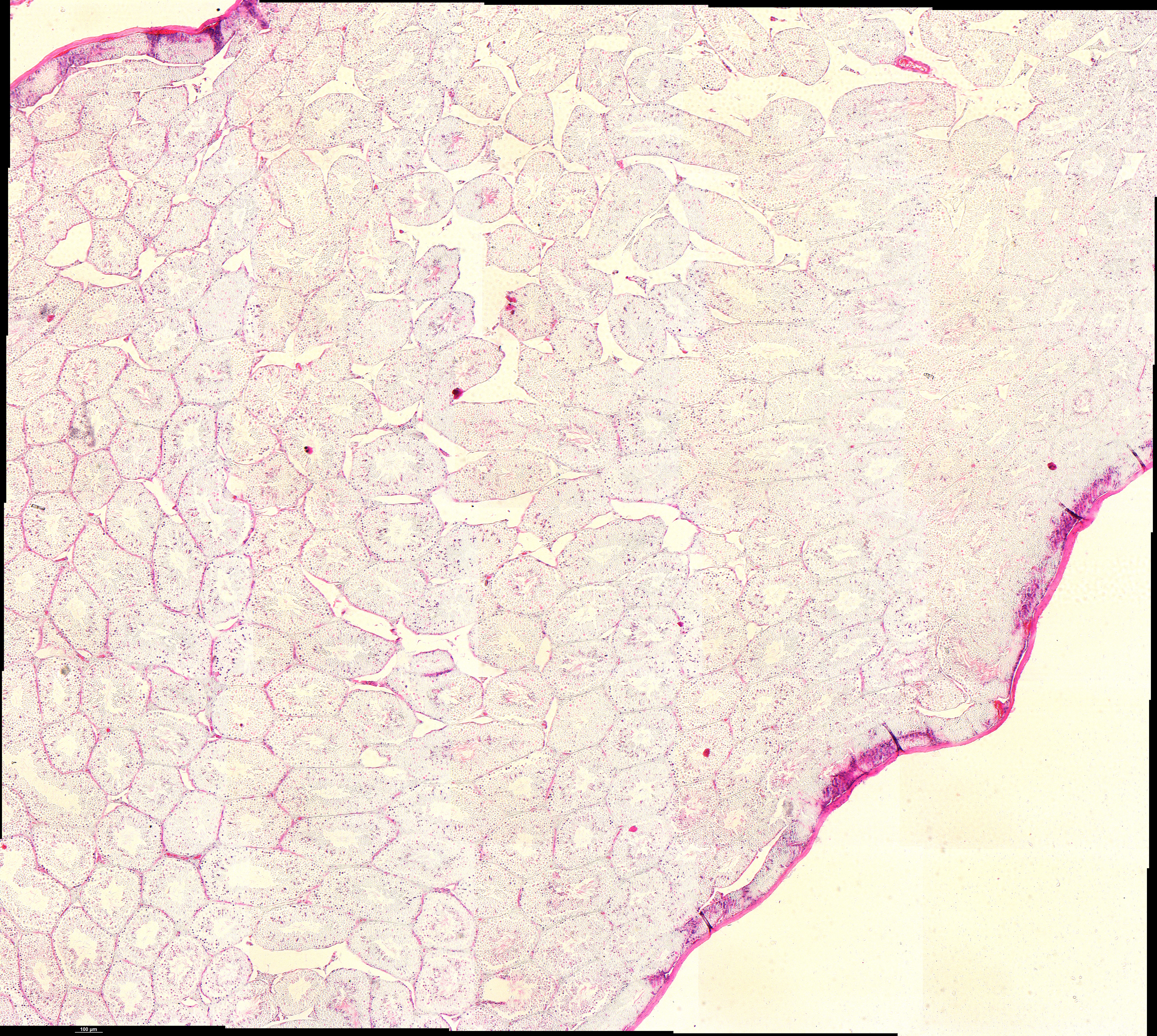

Supplement: Supplementary file 3 — Source data Fig. 1 [file 44321_2025_369_MOESM3_ESM.zip › Figure 1/Figure 1E/Figure 1E.PNG]

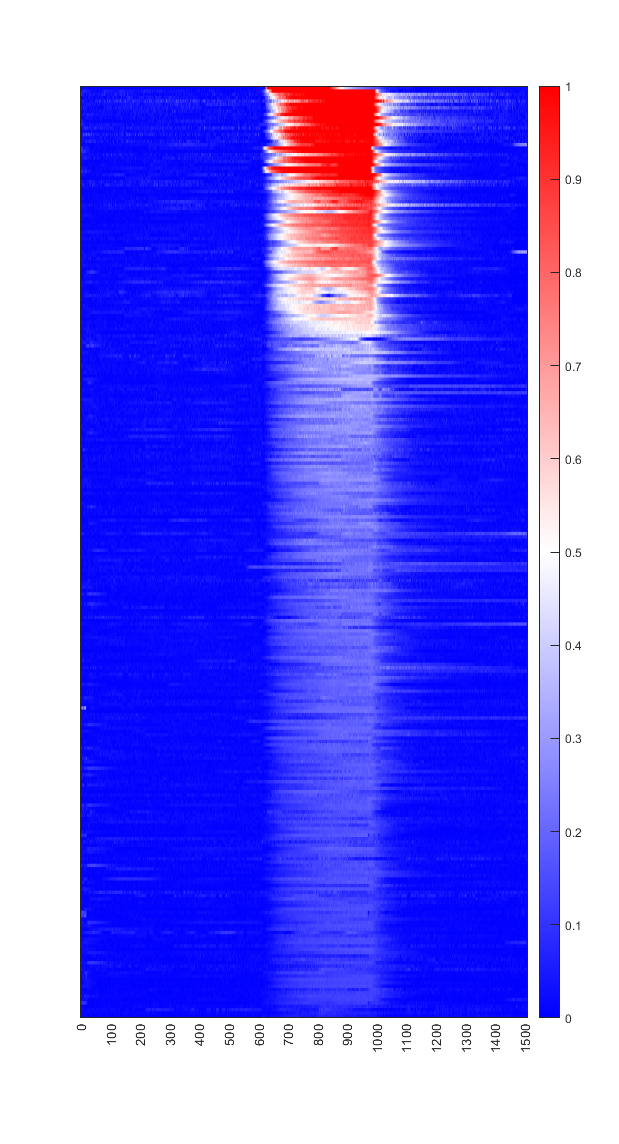

Supplement: Supplementary file 4 — Source data Fig. 2 [file 44321_2025_369_MOESM4_ESM.zip › Figure 2/Figure 2A/Figure 2A.tif]

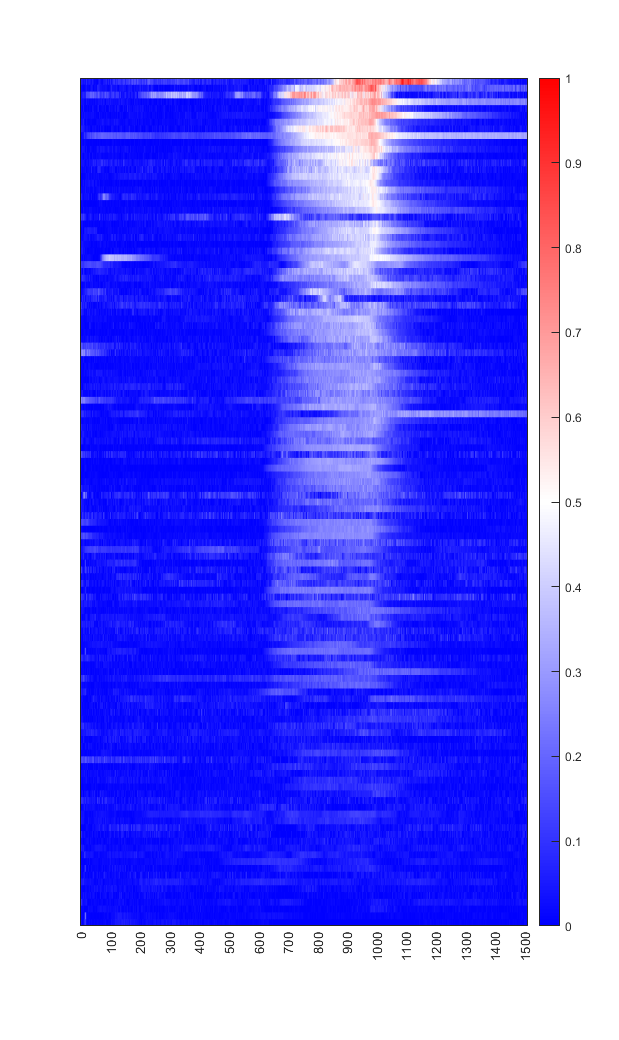

Supplement: Supplementary file 4 — Source data Fig. 2 [file 44321_2025_369_MOESM4_ESM.zip › Figure 2/Figure 2B/Figure 2B.tif]

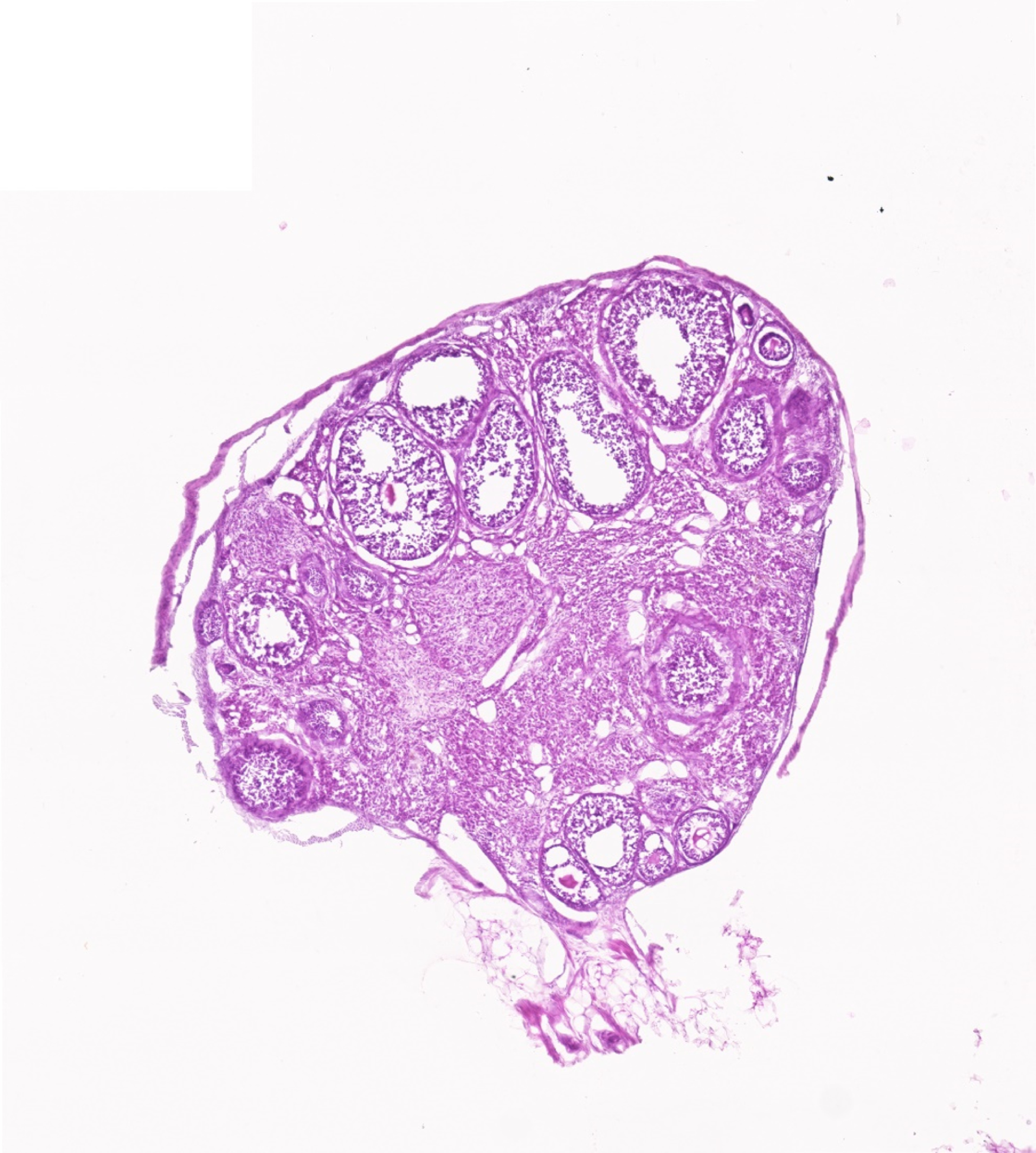

Supplement: Supplementary file 6 — Source data Fig. 5 [file 44321_2025_369_MOESM6_ESM.zip › Figure 5/Figure 5D/Figure 5D.tif]

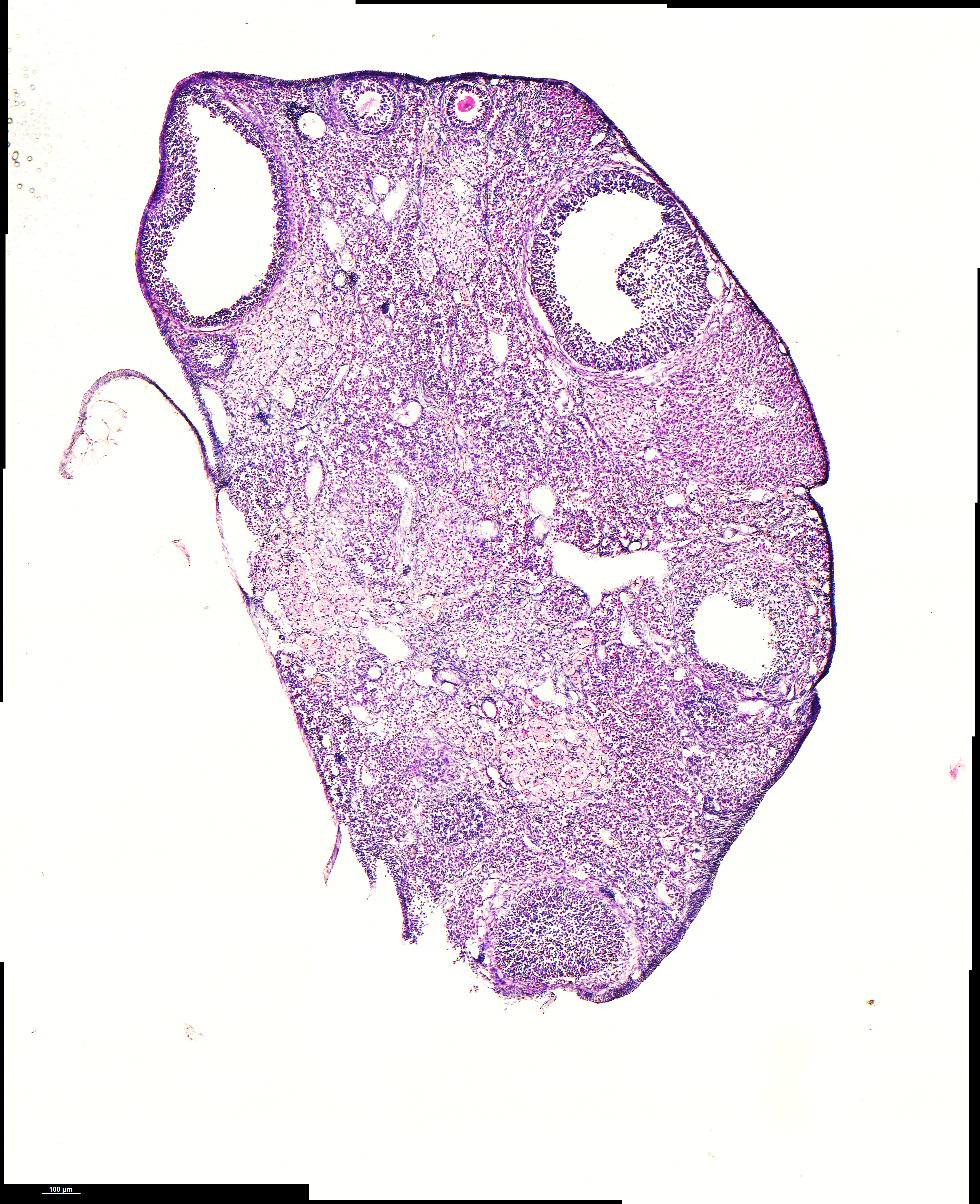

Supplement: Supplementary file 6 — Source data Fig. 5 [file 44321_2025_369_MOESM6_ESM.zip › Figure 5/Figure 5E/Figure 5E.tif]

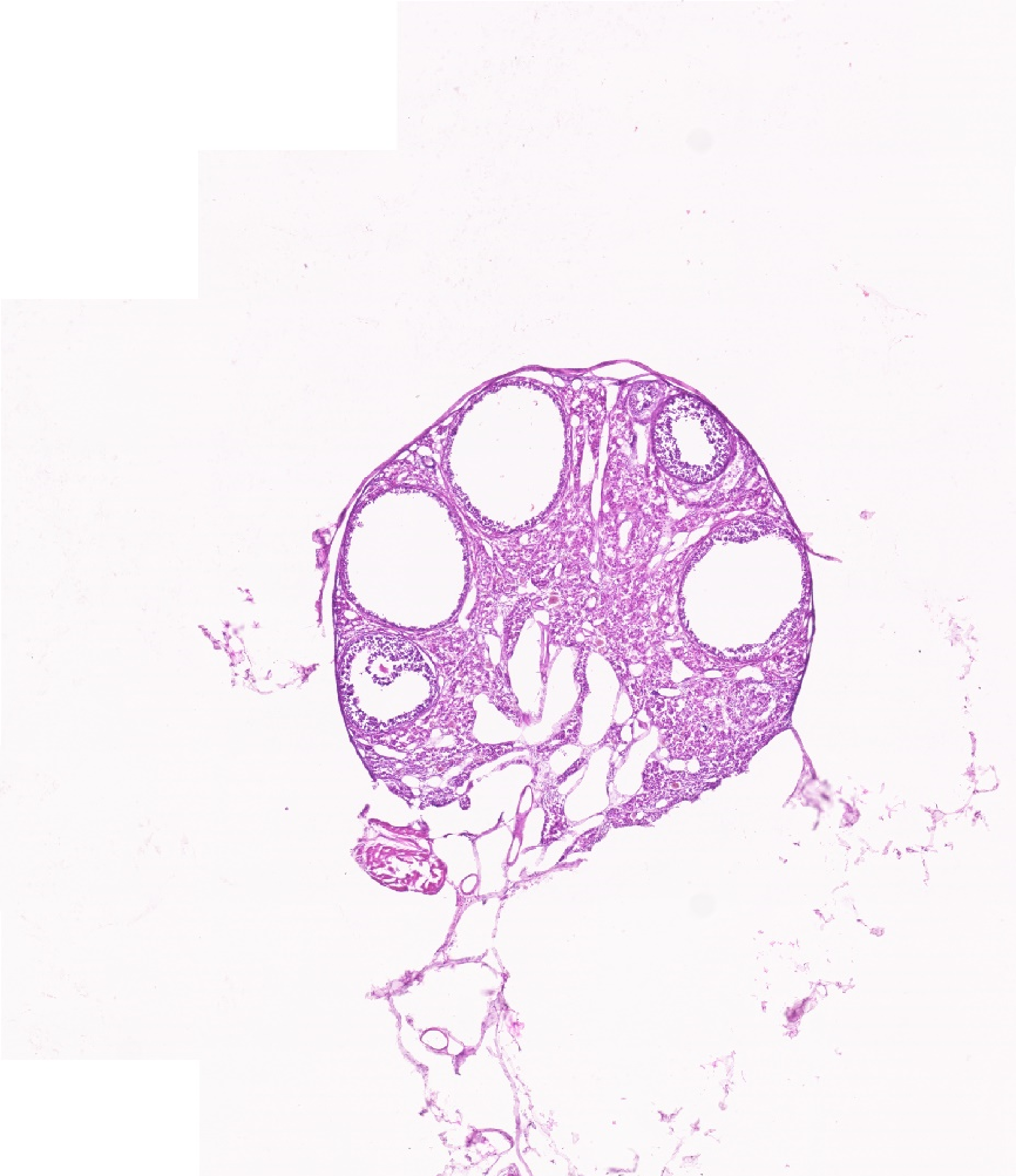

Supplement: Supplementary file 6 — Source data Fig. 5 [file 44321_2025_369_MOESM6_ESM.zip › Figure 5/Figure 5F/Figure 5F.tif]

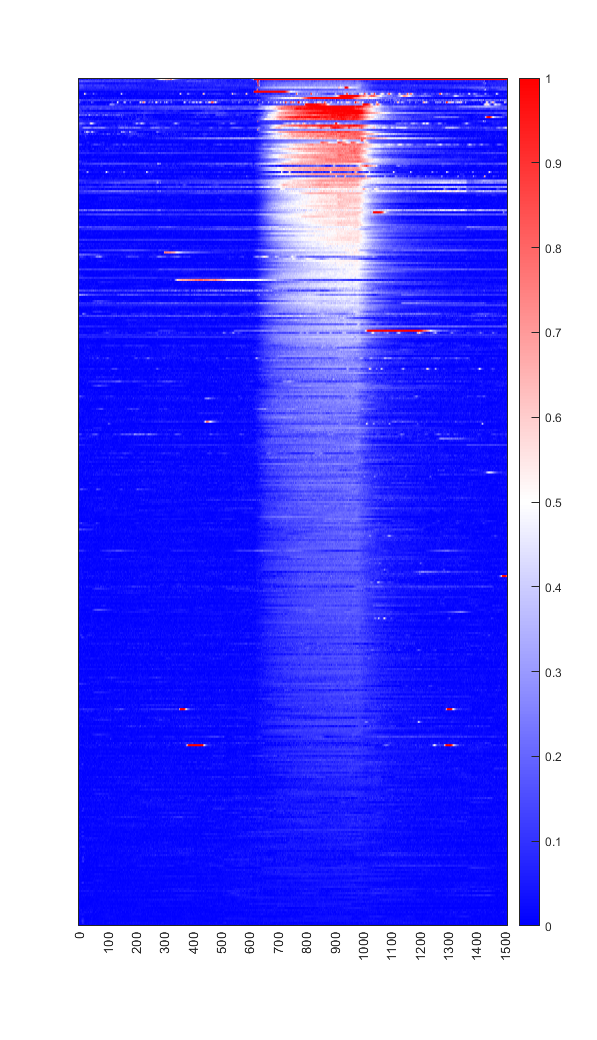

Supplement: Supplementary file 7 — Source data Fig. 6 [file 44321_2025_369_MOESM7_ESM.zip › Figure 6/Figure 6A/Figure 6A.tif]

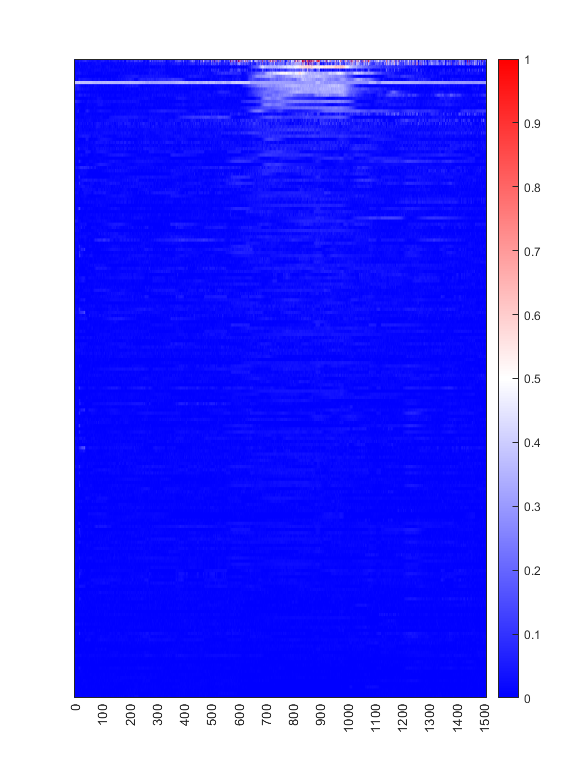

Supplement: Supplementary file 7 — Source data Fig. 6 [file 44321_2025_369_MOESM7_ESM.zip › Figure 6/Figure 6B/Figure 6B.tif]

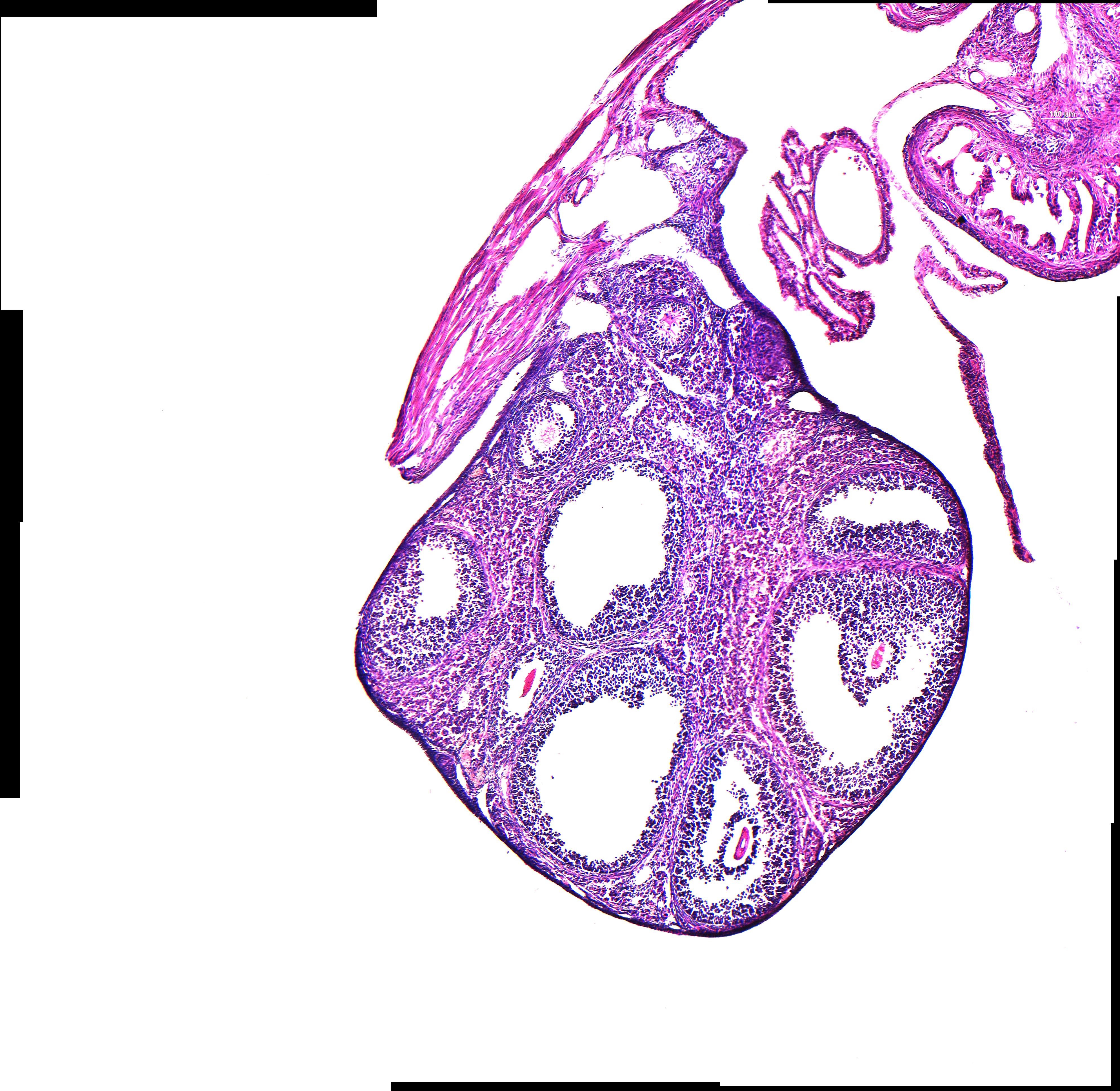

Supplement: Supplementary file 8 — Source data Fig. 8 [file 44321_2025_369_MOESM8_ESM.zip › Figure 8/Figure 8A/Figure 8A.PNG]

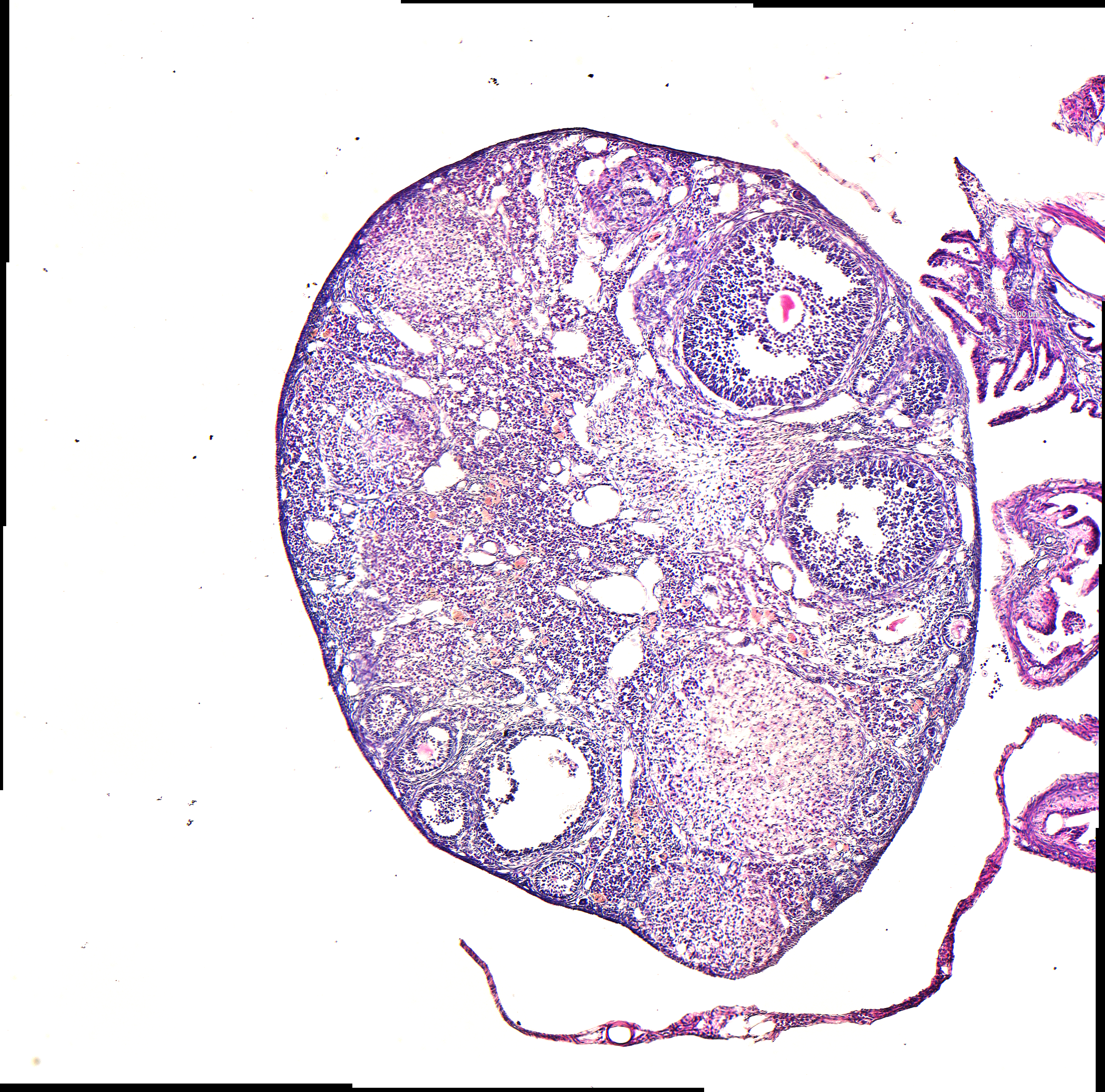

Supplement: Supplementary file 8 — Source data Fig. 8 [file 44321_2025_369_MOESM8_ESM.zip › Figure 8/Figure 8B/Figure 8B.PNG]

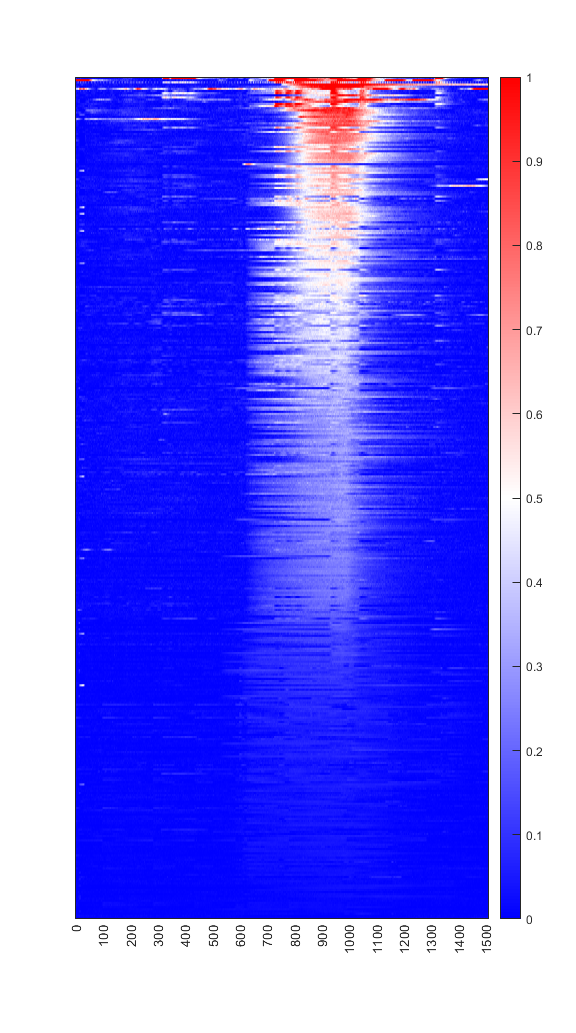

Supplement: Supplementary file 9 — Source data Fig. 9 [file 44321_2025_369_MOESM9_ESM.zip › Figure 9/Figure 9A/Figure 9A.tif]

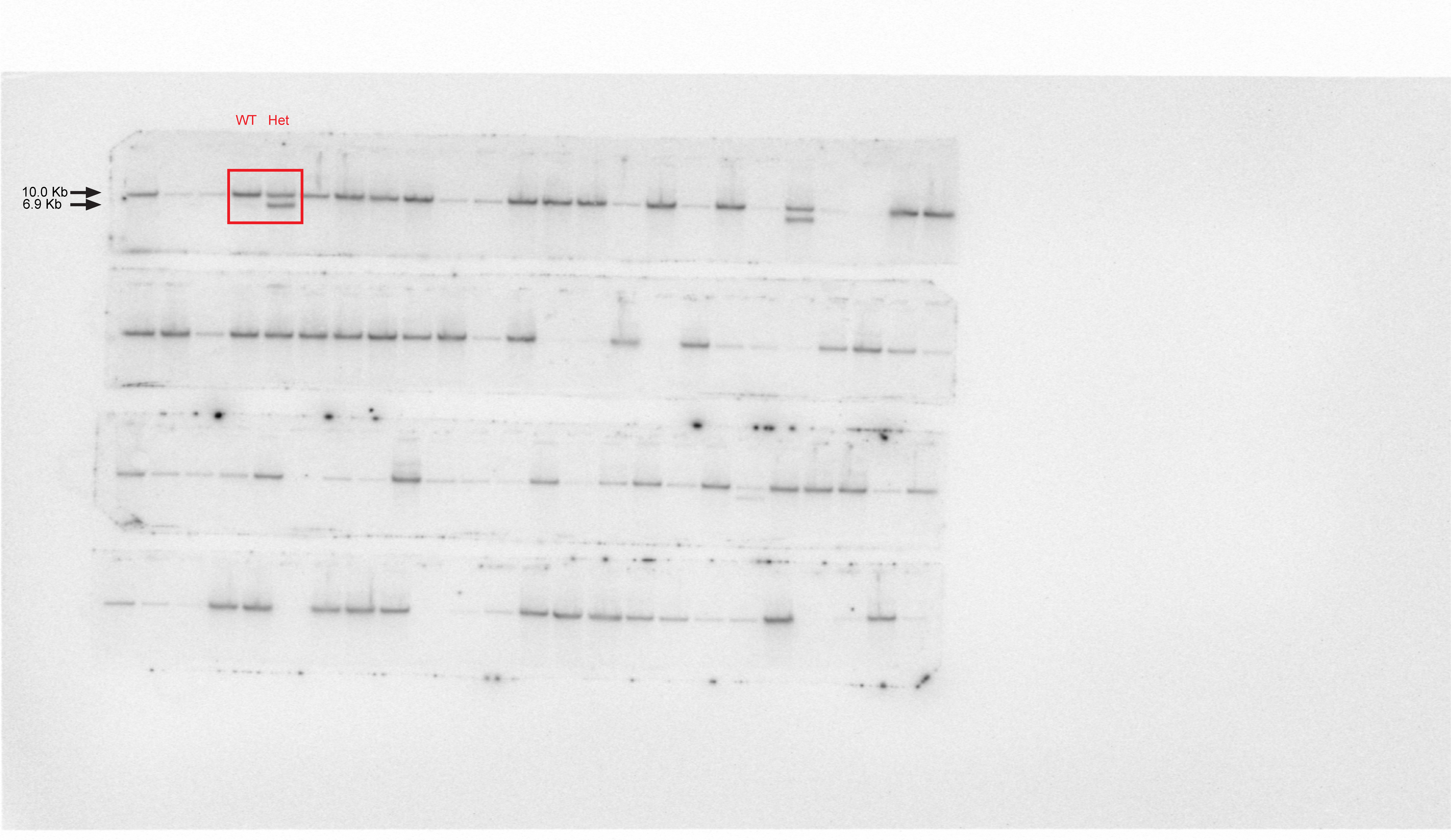

Supplement: Supplementary file 10 — Figure EV1 Source Data [file 44321_2025_369_MOESM10_ESM.zip › Figure EV1/Figure EV1B/Figure EV1B.tif]
