# Supplementary material for: Functional rescue and AI analysis of a human inactivating GPCR mutation using a small molecule
Source: EMBO Mol Med. 2026 Jan 8;18(2):725–58. doi: 10.1038/s44321-025-00369-2 (PMC12905377; doi:10.1038/s44321-025-00369-2)
Supplement: Supplementary file 12 — Expanded View Figures [file 44321_2025_369_MOESM12_ESM.pdf]

Expanded View Figures

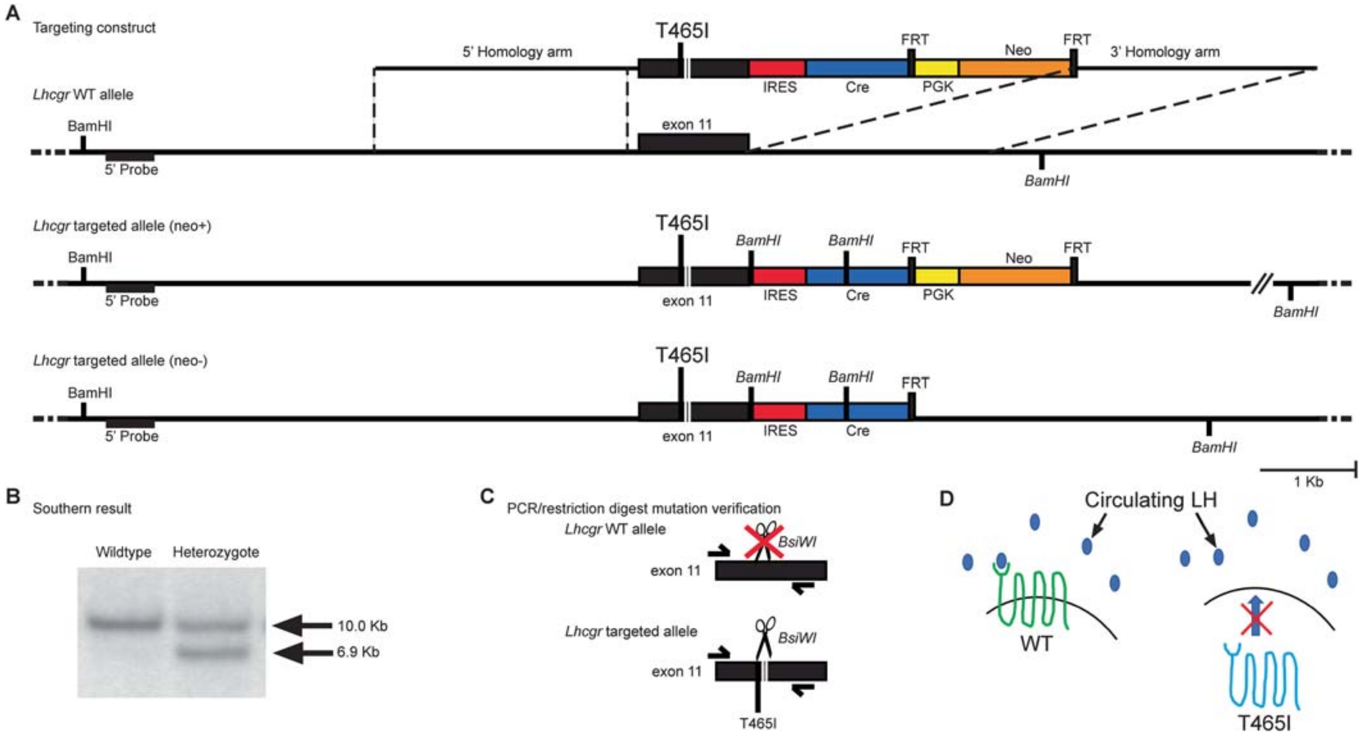

Figure EV1. A novel mouse strain harboring the LHRT465I mutation and driving Cre recombinase expression under the control of the *Lhcgr* promoter.

(**A**) A schematic diagram of the targeting construct used for the generation of the LHRT465I-IC$^{+/-}$ mouse model. The targeting construct carries the T465I mutation in exon 11, followed by an IRES and a Cre recombinase-encoding sequence (Candlish et al, 2015). (**B**) Southern blot showing the two bands obtained for the heterozygote mouse upon cleavage of the targeted allele by Bam*HI*. (**C**) A Bsi*WI* restriction digestion site adjacent to the T465I mutation was introduced into exon 11 to confirm its integration in the targeted allele. (**D**) A schematic diagram of the effect of the T465I mutation. The wild-type (WT) LHR is a G protein-coupled receptor that is targeted to the cell membrane, where it can bind circulating LH. However, the T465I mutation impairs recruitment of the mutant LHR to the cell membrane, preventing it from binding and responding to circulating LH. LH luteinizing hormone, IRES internal ribosomal entry site. Source data are available online for this figure.

 © The Author(s)

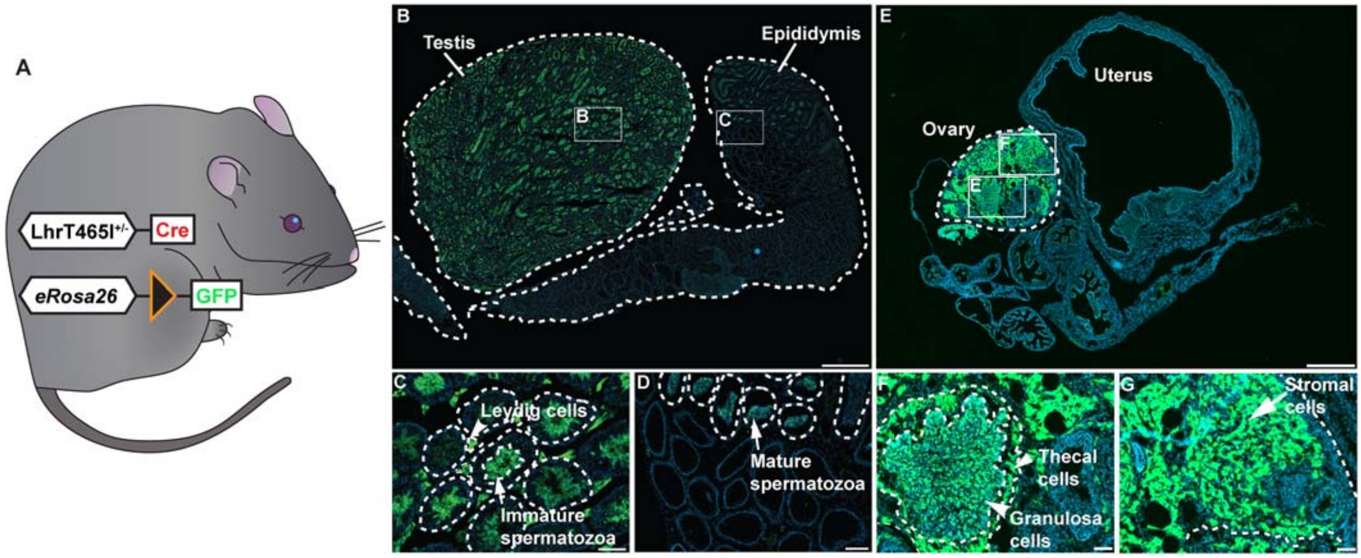

Figure EV2. Reporter gene expression in the reproductive organs of the LhrT465I-IC$^{+/-}$/eR26-GFP mice.

(**A**) LhrT465I-IC$^{+/-}$/eR26-GFP mice were generated by crossing LhrT465I-IC$^{+/-}$ mice with eR26-GFP mice. In these mice, all cells expressing *Lhcgr* also express Cre, which excises the stop signal upstream of GFP. Hence, all cells expressing the luteinizing hormone receptor (LHR) also express GFP. (**B**) Representative image of a testis from a LhrT465I-IC$^{+/-}$/eR26-GFP mouse showing GFP expression in the Leydig cells and spermatozoa. Scale bar: 2000 µm. (**C**) Inset showing GFP expression in Leydig cells (triangle arrow) and mature spermatozoa (arrow) inside seminiferous tubules. Scale bar: 200 µm. (**D**) Inset showing GFP expression in immature spermatozoa (arrow) inside the epididymis. Scale bar: 200 µm. (**E**) Representative image of an ovary from a LhrT465I-IC$^{+/-}$/eR26-GFP mouse showing GFP expression in the granulosa, thecal and stromal cells. Scale bar: 500 µm. (**F**) Inset showing GFP expression in granulosa (triangle arrow) and thecal (small triangle arrow) cells. Scale bar: 50 µm. (**G**) Inset showing GFP expression in stromal cells. Scale bars: 50 µm.

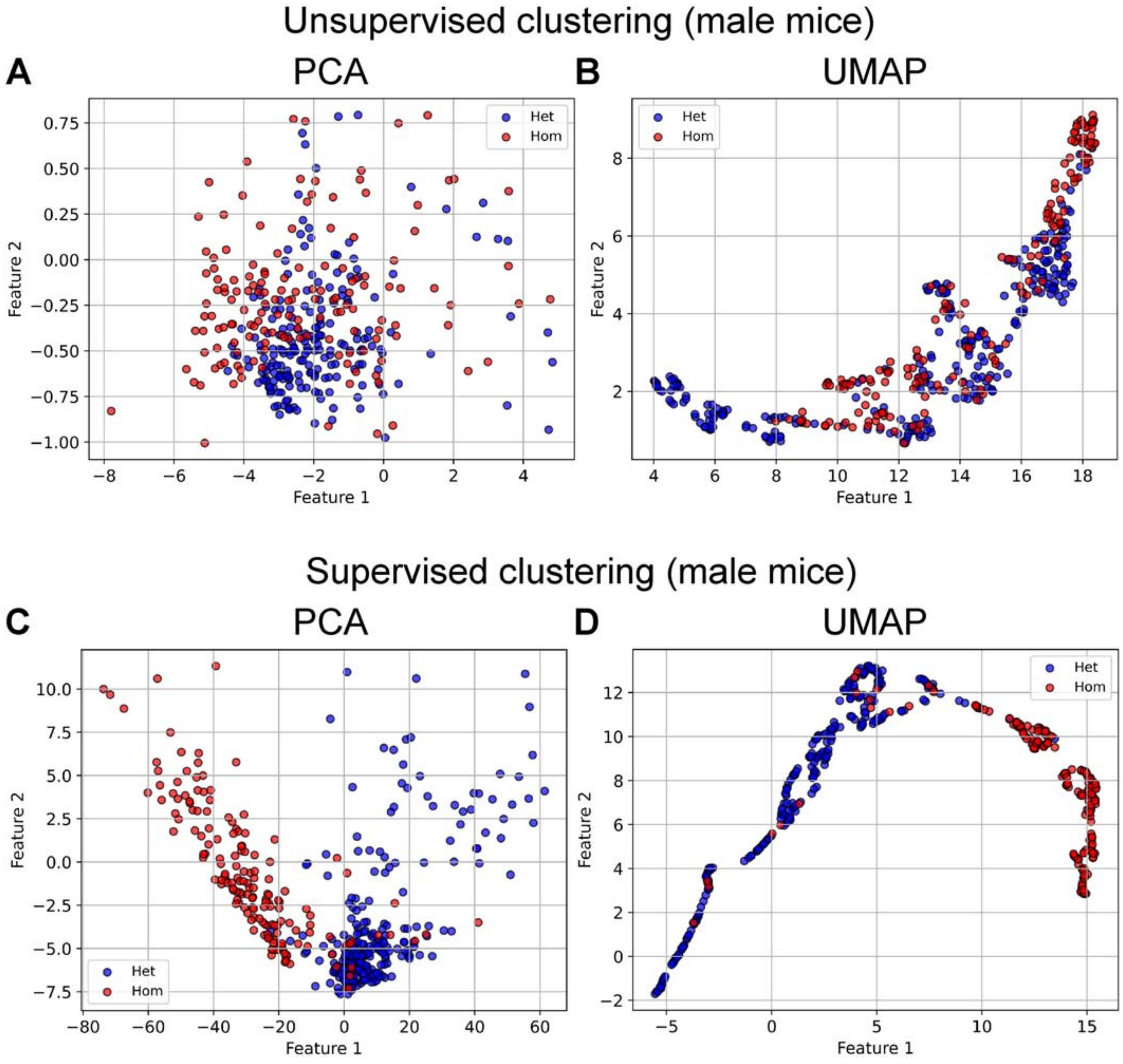

Figure EV3. PCA and UMAP plots for visualizing the distribution of Leydig cells based on their calcium profiles.

(**A**) A PCA plot for unsupervised clustering (without AI), which shows patterns in the direction of highest variance, does not clearly distinguish between Leydig cells from control Het and mutant Hom male mice. (**B**) A UMAP plot for unsupervised clustering, which best preserves the true global geometry, is slightly better at distinguishing between Het and Hom cells, with some small subclusters beginning to emerge. (**C**) PCA representation of supervised clustering (AI model) shows two distinct clusters, predominantly composed of control Het or mutant Hom cells. (**D**) UMAP representation of supervised clustering shows a narrower distribution of cells, with tighter, largely pure subclusters.

 © The Author(s)

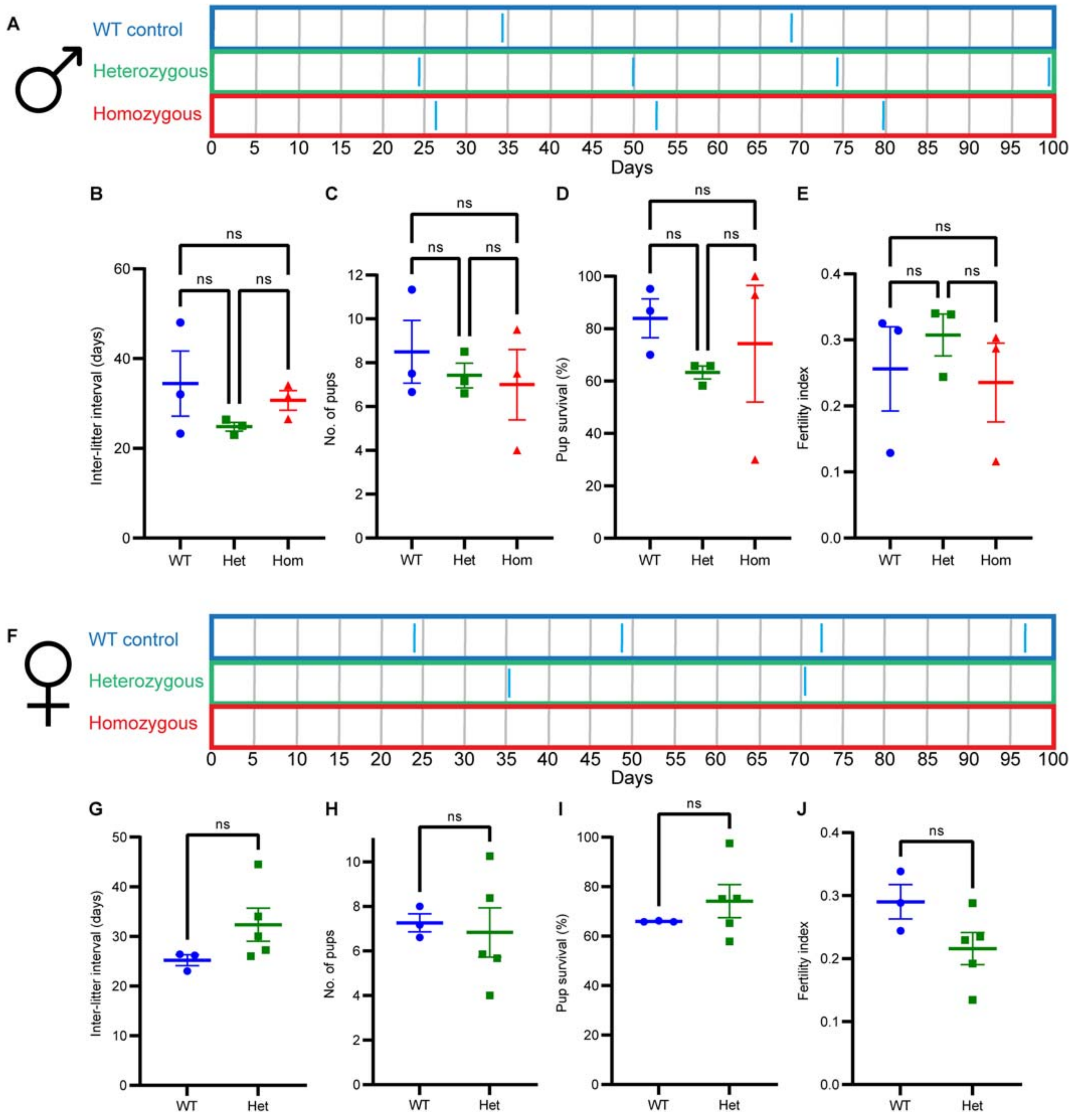
A
WT control
Heterozygous
Homozygous
Days
B
Inter-litter interval (days)
C
No. of pups
D
Pup survival (%)
E
Fertility index
ns
WT
Het
Hom
F
WT control
Heterozygous
Homozygous
Days
G
Inter-litter interval (days)
H
No. of pups
I
Pup survival (%)
J
Fertility index
WT
Het

Figure EV4. Homozygous mutant females (LhrT465I-IC$^{+/+}$), but not males, are infertile.

(**A**) Breeding pairs were set up for WT control (LhrT465I-IC$^{-/-}$), heterozygous (Het) (LhrT465I-IC$^{+/-}$), and homozygous (Hom) male mice with one WT female mouse each; representative breeding pairs (with WT mate) have been shown, with blue ticks marking the birth of a litter. Based on the number of days elapsed before the birth of each litter, all three groups showed comparable fertility. (**B**) Time elapsed between two litters (or until birth of the first one) was plotted as inter-litter interval. ns indicates lack of statistical significance ($P = 0.3384$ for WT vs. Het, $P = 0.8246$ for WT vs. Hom, $P = 0.6369$ for Het vs. Hom). (**C**) Average number of pups born per litter was similar across groups ($P = 0.8292$ for WT vs. Het, $P = 0.7029$ for WT vs. Hom, $P = 0.9709$ for Het vs. Hom). (**D**) The survival rate of pups was also not significantly different across the groups ($P = 0.5622$ for WT vs. Het, $P = 0.8719$ for WT vs. Hom, $P = 0.8404$ for Het vs. Hom). (**E**) Fertility index is a composite measure considering the rate of birth of pups and litter size; this index was also similar across groups ($P = 0.7842$ for WT vs. Het, $P = 0.9604$ for WT vs. Hom, $P = 0.6318$ for Het vs. Hom). (**F**) Breeding pairs were set up for WT control, heterozygous, and homozygous female mice with one WT (or Het) male mouse each; representative breeding pairs have been shown. WT and Het females exhibited similar breeding success, whereas the Hom mutant female mice were infertile. (**G**) Inter-litter interval was not significantly different between WT and Het females ($P = 0.1638$). (**H**) The average number of pups per litter was not significantly different between WT and Het females ($P = 0.7863$). (**I**) Survival rate of pups was not significantly different between WT and Het females ($P = 0.3909$). (**J**) The composite fertility index, although showing a downward trend for Het females compared to WT females, was not significantly different between the two groups ($P = 0.1076$). Data information: In (**B–E**), data were presented as mean ± SEM. $^*P < 0.05$ (ANOVA). $N = 3$ mice per group. In (**G–J**), data were presented as mean ± SEM. $^*P < 0.05$ (two-tailed unpaired Student's *t*-test). $N = 3$ (WT) and 5 (Het). Source data are available online for this figure.

 © The Author(s)

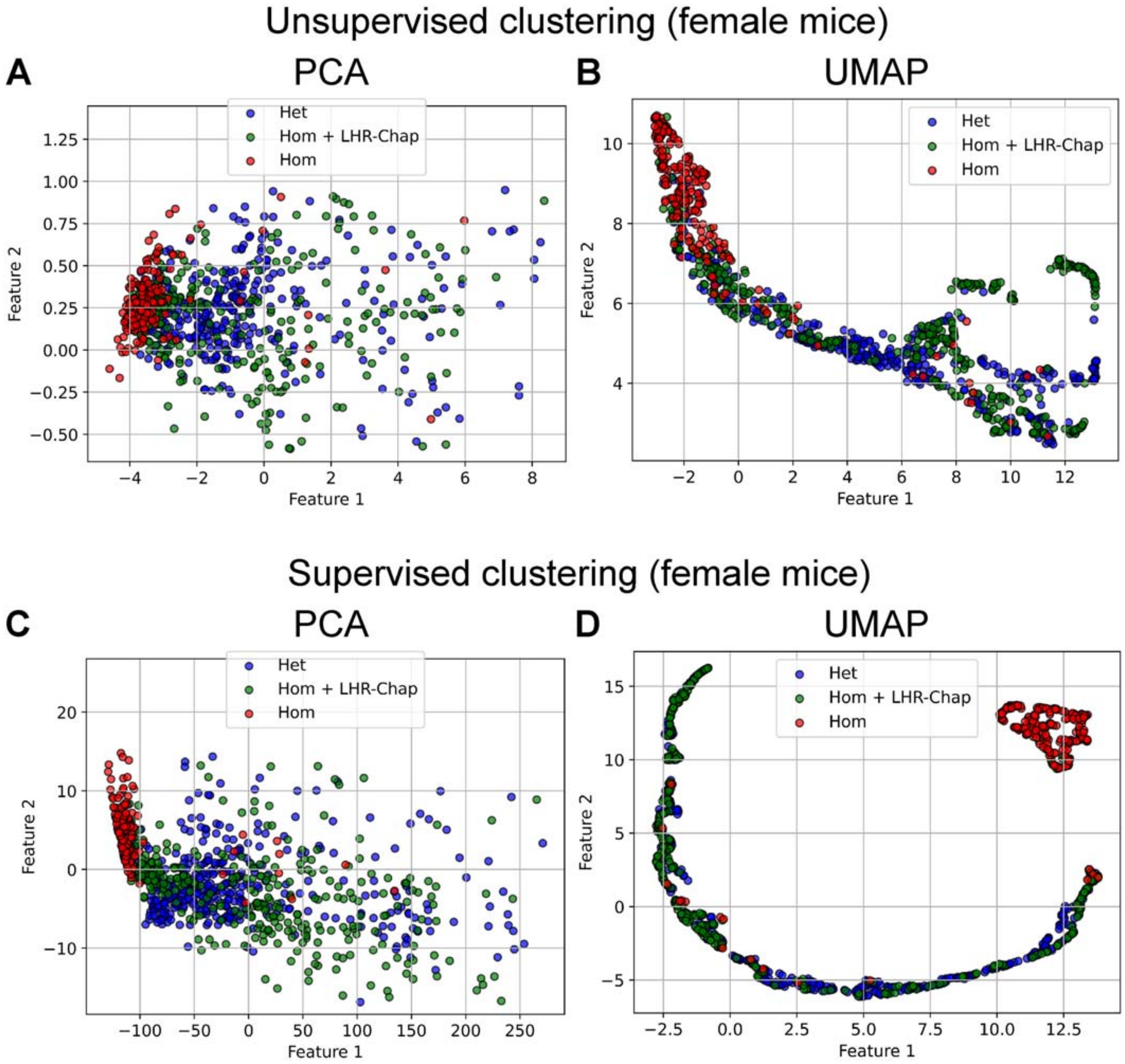

Figure EV5. PCA and UMAP plots for visualizing the distribution of ovarian cells based on their calcium profiles.

(**A**) A PCA plot of unsupervised clustering (without AI) showed mutant Hom cells being restricted largely to one side of the plot, although with considerable overlap with both control Het and LHR-Chap-treated Hom ovarian cells, which were both more widely dispersed. Beyond this, no other meaningful subclusters could be detected. (**B**) A UMAP plot showed a more restricted distribution of cells from all three groups, with small subclusters emerging. A minor overlap was observed between the Hom and LHR-Chap-treated Hom cells, which otherwise clustered closer to the control Het cells. (**C**) PCA plot of AI-based supervised clustering shows a much tighter clustering of the Hom cells, overlapping very little with cells from the other two groups; control Het and LHR-Chap-treated Hom ovarian cells were distributed close to each other, with few distinct clusters. (**D**) UMAP representation of supervised clustering revealed a tight cluster of Hom cells, which were well-separated from control and treated cells. Subclusters composed mostly of either control or treated cells indicate that the treatment does not restore the mutant cells to exact control conditions, achieving imperfect functional rescue that approximates the calcium profiles of the control cells.

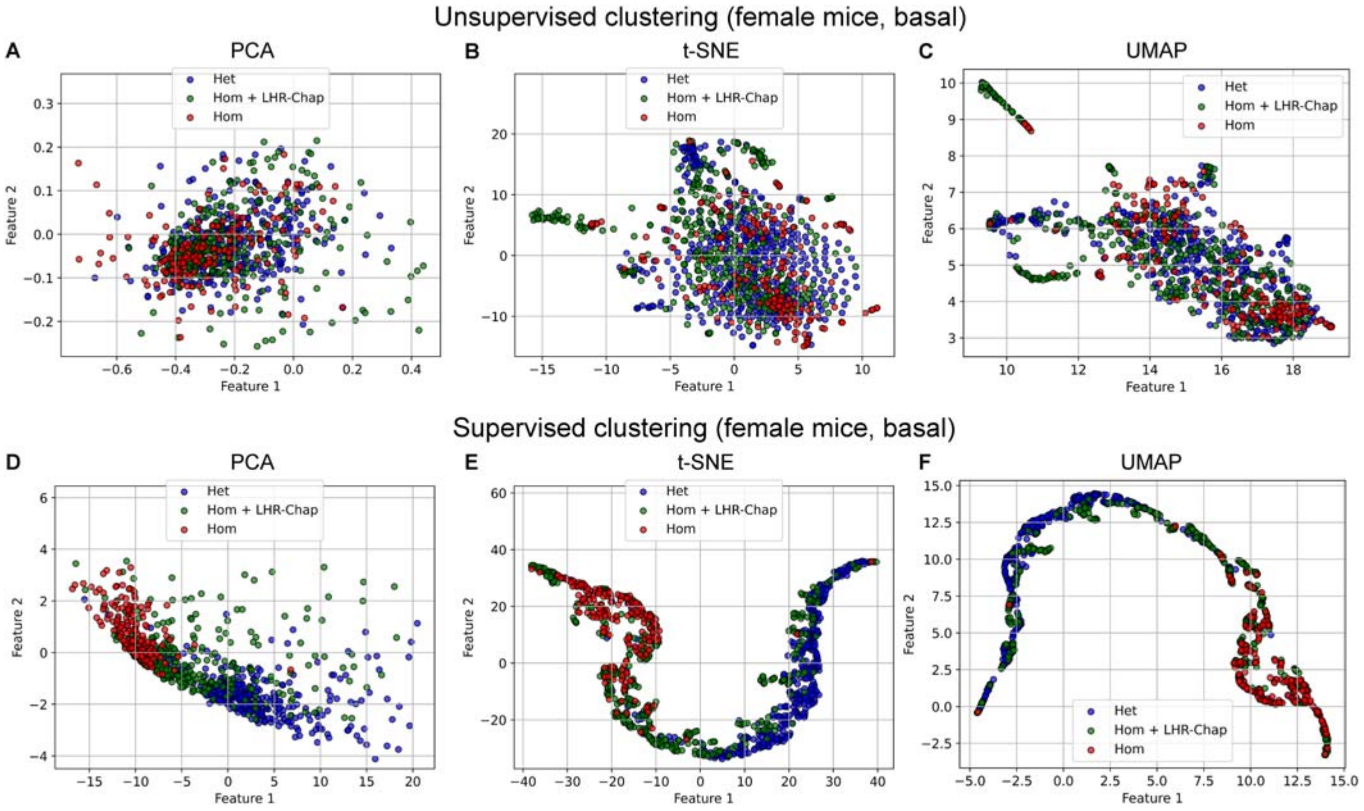

Figure EV6. AI can distinguish Het from Hom cells, and assess functional impact of treatment, based on spontaneous (basal) calcium profiles alone.

(**A–C**) Visualization of unsupervised clustering of ovarian cells based on spontaneous calcium signals (corresponding to "basal" section of calcium imaging) using PCA (**A**), t-SNE (**B**), and UMAP (**C**) approaches did not reveal meaningful clusters. (**D**) Supervised (AI model) clustering based on spontaneous calcium signals revealed a clearly skewed distribution pattern, even with PCA. (**E**, **F**) t-SNE (**E**) and UMAP (**F**) visualization show a clear segregation of untreated mutant Hom cells from control Het and LHR-Chap-treated Hom cells, showcasing the ability of the feature-learning model to tease apart these groups based on distinctive spontaneous calcium signaling patterns alone.

 © The Author(s)
